# Antibody-Dependent Cellular Cytotoxicity-Competent Antibodies against HIV-1-Infected Cells in Plasma from HIV-Infected Subjects

**DOI:** 10.1128/mBio.02690-19

**Published:** 2019-12-17

**Authors:** Franck P. Dupuy, Sanket Kant, Alexandre Barbé, Jean-Pierre Routy, Julie Bruneau, Bertrand Lebouché, Cécile Tremblay, Marzena Pazgier, Andrés Finzi, Nicole F. Bernard

**Affiliations:** a Research Institute of the McGill University Health Centre (RI-MUHC), Montreal, Quebec, Canada; b Infectious Diseases and Immunity in Global Health Program, RI-MUHC, Montreal, Quebec, Canada; c Division of Experimental Medicine, McGill University, Montreal, Quebec, Canada; d Division of Hematology, McGill University Health Centre, Montreal, Quebec, Canada; e Chronic Viral Illness Service, McGill University Health Centre, Montreal, Quebec, Canada; f Department of Family Medicine, Université de Montréal, Montreal, Quebec, Canada; g Centre de Recherche du Centre Hospitalier de l’Université de Montréal (CRCHUM), Montreal, Quebec, Canada; h Department of Family Medicine, McGill University, Montreal, Quebec, Canada; i Département de Microbiologie, Infectiologie et Immunologie, Université de Montréal, Montreal, Quebec, Canada; j Infectious Diseases Division, Department of Medicine of Uniformed Services, University of the Health Sciences, Bethesda, Maryland, USA; k Department of Microbiology and Immunology, McGill University, Montreal, Quebec, Canada; l Division of Clinical Immunology, McGill University Health Centre, Montreal, Quebec, Canada; McMaster University

**Keywords:** broadly neutralizing antibodies, HIV Envelope, ADCC, ADCC assay, CD4 binding site antibodies, CD4i antibodies, neutralizing antibodies

## Abstract

Measuring Envelope (Env)-specific antibody (Ab)-dependent cellular cytotoxicity (ADCC)-competent Abs in HIV^+^ plasma is challenging because Env displays distinctive epitopes when present in a native closed trimeric conformation on infected cells or in a CD4-bound conformation on uninfected bystander cells. We developed an ADCC model which distinguishes Env-specific ADCC-competent Abs based on their capacity to eliminate infected, bystander, or Env rgp120-coated cells as a surrogate for shed gp120 on bystander cells. A panel of monoclonal Abs (MAbs), used to opsonize these target cells, showed that infected cells were preferentially recognized/eliminated by MAbs to CD4 binding site, V3 loop, and viral spike epitopes whereas bystander/coated cells were preferentially recognized/eliminated by Abs to CD4-induced (CD4i) epitopes. In HIV-positive (HIV^+^) plasma, Env-specific Abs recognized and supported ADCC of infected cells, though a majority were directed toward CD4i epitopes on bystander cells. For ADCC activity to be effective in HIV control, ADCC-competent Abs need to target genuinely infected cells.

## Abstract (precis)

HIV Env-specific nonneutralizing Abs (NnAbs) able to mediate ADCC have been implicated in protection from HIV infection. However, Env-specific NnAbs have the capacity to support ADCC of both HIV-infected and HIV-uninfected bystander cells, potentially leading to misinterpretations when the assay used to measure ADCC does not distinguish between the two target cell types present in HIV cultures. Using a novel ADCC assay, which simultaneously quantifies the killing activity of Env-specific Abs on both infected and uninfected bystander cells, we observed that only a minority of Env-specific Abs in HIV^+^ plasma mediated ADCC of genuinely HIV-infected cells displaying Env in its native closed conformation. This assay can be used for the development of vaccine strategies aimed at eliciting Env-specific Ab responses capable of controlling HIV infection.

## INTRODUCTION

The RV144 Thai trial was the first and only HIV vaccine trial to date to show moderate (31%) but significant efficacy at protecting against HIV infection (1). Protection was not associated with the presence of broadly neutralizing antibodies (BnAbs) or cytotoxic T cell responses (2). Results from analyses of correlates of protection suggested that protection was associated with the induction of nonneutralizing immunoglobulin G (IgG) Abs directed to the V1/V2 loop of HIV Envelope (Env) gp120 (2–4). Also reported to be associated with protection from infection were Env-specific IgG nonneutralizing Abs (NnAbs) able to mediate Ab-dependent cellular cytotoxicity (ADCC) provided that no competing IgA Abs were present (2, 5–7). This has led to heightened interest in describing the determinants of effective anti-HIV directed ADCC activity.

HIV Env glycoprotein is the HIV gene product targeted by ADCC-competent Abs since it is the only viral protein exposed on the surface of infected cells (8). Env is a trimer assembled of heterodimers constituted of gp120 and gp41 glycoproteins. Whereas gp120 forms the outer part of the trimer, gp41 is largely buried at the trimer interface and anchors Env on the plasma membrane (9–12). Unliganded Env is normally present in a “closed” conformation on the surface of virions and infected cells (13, 14). Env interaction with CD4 drives the transition of a closed Env conformation to a CD4-bound “open” conformation (3, 4, 13). CD4 binding to gp120 occurs mainly during the attachment of viral particles to CD4^+^ target cells (T) at viral entry, as CD4 is downregulated from the surface of productively infected cells by Nef and Vpu (4, 15). However, gp120 is reported to shed from the surface of infected cells and to bind to CD4 on uninfected bystander cells, which then display Env in an open conformation (16, 17).

The CD4-bound Env conformation was proposed to represent a preferential target for ADCC-competent Abs present in HIV^+^ plasma (4, 14, 15). In its open conformation, Env exposes CD4-induced (CD4i) epitopes in the cluster A region (4, 18, 19), a conserved part of the gp120 inner domain hidden when Env is in a closed conformation (4, 15, 18, 20–22). CD4i epitopes are recognized by an important class of nonneutralizing ADCC-competent Abs (4, 15, 16, 23), which also bind gp120 shed from HIV-infected cells and taken up by bystander cells (15–17).

A frequently used ADCC target cell is the CEM.NKR.CCR5 (CEM) cell line coated with monomeric recombinant gp120 (rgp120) (24, 25). CEM cells are resistant to direct natural killer (NK) cell cytolysis (26, 27). Just as shed gp120 binds bystander cells, rgp120 coats CEM target cells through CD4 interactions, forcing gp120 to assume the CD4-bound conformation recognized by anti-cluster A monoclonal antibodies (MAbs) such as prototypical A32 (17, 28, 29). HIV-infected CEM cells and primary CD4 cells have also been used as ADCC targets (16, 30–32). HIV infection of CD4^+^ T cells usually results in only a fraction becoming infected. Uninfected CD4^+^ bystander cells coated with shed gp120 or virions present in the viral inoculum expose CD4i epitopes, which trigger the recognition and killing of bystander cells through ADCC (16, 33). This compromises the measurement of Env-specific ADCC-competent Abs capable of mediating the killing of genuinely infected cells. Other studies evaluating the presence of ADCC-competent Abs in HIV^+^ plasma have used HIV isolates presenting Nef and/or Vpu deletions or having mutations in these gene products that compromise their ability to downmodulate cell surface CD4, making it available to bind Env, which then assumes an open conformation recognized by anti-cluster A MAbs (25, 34). The use of rgp120-coated cells, partially HIV-infected cells, or cells infected with a virus unable to downregulate CD4 as ADCC target cells is the basis for the widely held view that Abs to CD4i epitopes dominate the HIV Env-specific ADCC-competent Ab pool in plasma from HIV-infected subjects (25, 35).

We describe here the generation of a CEM target cell that is close to 100% HIV infected as determined by intracellular p24 staining. The virus used to infect CEM cells codes for HIV Bal Env and expresses wild-type Nef and Vpu able to downregulate CD4, exposing Env in a closed conformation. A panel of BnAbs, NnAbs, and control Abs was used to probe the epitope structure of cell surface Env on infected CEM cells in comparison to bystander and rgp120-coated CEM (cCEM) cells. The ability of each Ab to support the ADCC activity of the three target cells was assessed. We also describe a novel assay designed to measure ADCC activity using the frequency of annexin V^+^ (AnV^+^) target cells as a readout and to compare the performance of this assay with that of the previously described ADCC-GranToxiLux assay (24). We confirm that anti-cluster A MAbs mediate ADCC of CEM cells coated with rgp120 or of bystander cells that have taken up shed gp120 or bound defective HIV virions but not of genuinely HIV-infected CEM cells. Most ADCC-competent Abs in HIV^+^ plasma recognize and kill target cells, exposing open-Env-conformation. Abs to epitopes other than CD4i epitopes that recognize and support ADCC of infected CEM cells expressing closed-conformation Env are also present in HIV^+^ plasma.

## RESULTS

### ADCC quantification of the frequency of annexin V (AnV)^+^ target cells.

**FIG 1:**
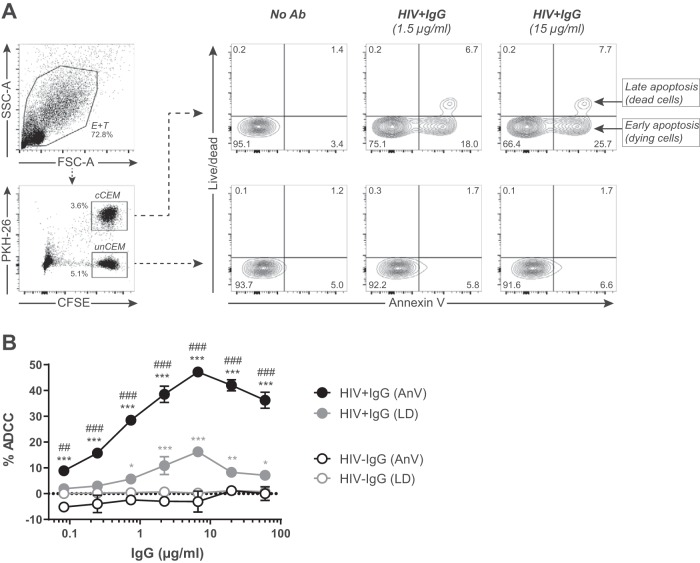
Flow-based measurement of ADCC activity using annexin V (AnV) staining of target cells. Uncoated CEM cells (unCEM) labeled with CFSE were mixed 1:1 with rgp120-coated CEM cells (cCEM) labeled with CFSE and PKH26 prior to the addition of opsonizing antibodies to be used as target (T) cells. Peripheral blood mononuclear cells (PBMCs) were used as effector (E) cells and mixed at an E:T ratio of 30:1. After 1 h of incubation, cells were stained with annexin V (AnV) and Live/Dead (LD) reagents to quantify the frequency of early/late apoptotic (AnV^+^) and dead (LD^+^) target CEM cells, respectively, by flow cytometry. (A) Gating strategy. Combined PBMC, cCEM cells, and unCEM cells were gated on by forward scatter A (FSC-A) and side scatter A (SSC-A). The frequencies (%) of AnV^+^ and LD^+^ CEM cells were evaluated among cCEM cells (CFSE^+^ PKH26^+^, upper right panel) and unCEM cells (CFSE^+^ PKH26^−^; lower right panel) cells opsonized with either 1.5 μg/ml (middle) or 15 μg/ml (right) of HIV^+^ IgG compared to no antibody (No Ig, left). Percentages of AnV^+^ and/or LD^+^ cells are indicated in each quadrant. (B) Dose-response curves showing ADCC activity (% ADCC) as measured by the percentage of AnV^+^ (black symbols) or LD^+^ (gray symbols) cCEM target cells opsonized with either HIV^+^ IgG (filled symbols) or HIV^−^ IgG (empty symbols) after background (No Ab) subtraction. Error bars indicate the standard deviation (s.d.) of replicates and significance was determined by comparing the percentages of ADCC between HIV^+^ IgG and HIV^−^ IgG for all IgG concentrations using AnV or LD (*, *P* < 0.05; **, *P* < 0.01; ***, *P* < 0.001) and by comparing the percentages of ADCC between the AnV^+^ and LD^+^ data for all HIV^+^ IgG concentrations (##, *P* < 0.01; ###, *P* < 0.001). Two-way ANOVA (Tukey’s multiple-comparison test) was used for both comparisons. Data represent results from one experiment representative of three.

Induction of apoptosis is the mechanism by which effector (E) cells kill Ab-opsonized target cells by ADCC. We designed a novel ADCC assay that measures levels of apoptotic target cells labeled with carboxyfluorescein succinimidyl ester (CFSE) with or without PKH26. This method employed AnV, a Ca2^+^-dependent protein with a high affinity for phosphatidylserines, to detect translocation of these molecules from the inner to the outer leaflet of the plasma membrane as a measure of levels of apoptotic cells. The gating strategy used to determine the frequency of AnV^+^ target cells generated in the ADCC-AnV assay employed peripheral blood mononuclear cells (PBMCs) as effector cells, CFSE^+^ PKH26^+^ coated CEM cells as target cells, and, as a negative control, CFSE^+^ PKH26^−^ uncoated CEM cells is shown in Fig. 1A. Target cells were opsonized, or not, with 1.5 and 15 μg/ml of HIV**^+^** IgG. Killing was dose-dependent and anti-Env specific since HIV^−^ IgG did not mediate ADCC (Fig. 1B). Using AnV to detect ADCC mediated by PBMC effector cells, a maximum of approximately 50% target cell killing was achieved with 6 μg/ml of HIV**^+^** IgG (Fig. 1B). By using a combination of AnV and Live/Dead (LD) cell staining, which detects dead cells with a compromised membrane, we showed that the AnV assay was more sensitive than Live/Dead staining for measuring ADCC since AnV detected both dying and dead cells (i.e., early and late apoptotic cells, respectively) following incubation with HIV**^+^** IgG whereas Live/Dead staining detected only dead cells (Fig. 1B).

**FIG 2:**
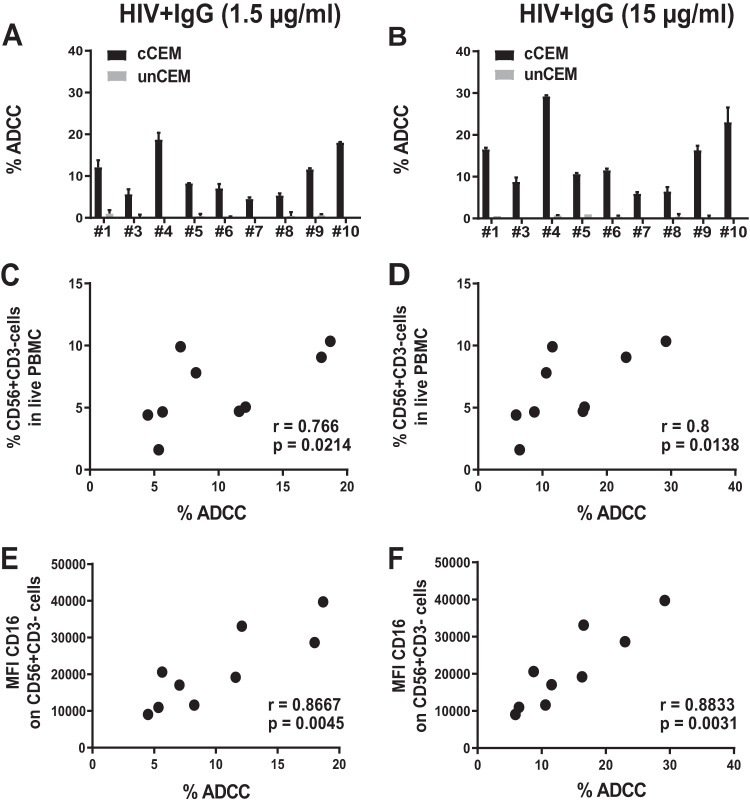
ADCC activity of effector PBMCs depends on the frequency of NK cells and CD16 levels on NK cells. Frequencies of NK cells (defined as live CD56^+^ CD3^−^) and levels of CD16 mean fluorescence intensity (MFI) on NK cells were evaluated in PBMCs from 9 HIV^−^ donors tested in parallel for their capacity to mediate ADCC of coated CEM cells (cCEM) and uncoated CEM (unCEM) cells opsonized with HIV^+^ IgG (as described for Fig. 1). (A nd B) Between-subject variation in the frequency of AnV^+^ (% ADCC) cCEM (black bars) and unCEM (gray bars) cells generated in the ADCC-AnV assay using PBMCs and 1.5 μg/ml (A) or 15 μg/ml (B) of HIV^+^ IgG to opsonize target cells. Data represent results from one experiment representative of three. Error bars indicate the standard deviations (SD) of results from replicates for each donor tested. (C and D) Spearman correlation between the frequency of NK cells present in live PBMC (% CD56^+^ CD3^−^ cells in live PBMC) and the frequency of AnV^+^ (% ADCC) cCEM cells opsonized with 1.5 μg/ml (C) or 15 μg/ml (D) of HIV^+^ IgG. (E and F) Spearman correlation between the MFI of CD16 expression on NK cells (MFI CD16 on CD56^+^ CD3^−^ cells) and the frequency of AnV^+^ (% ADCC) cCEM cells opsonized with 1.5 μg/ml (E) or 15 μg/ml (F) of HIV^+^ IgG.

PBMC effector cells from HIV-uninfected donors were tested in parallel for their capacity to mediate ADCC activity of coated CEM cells opsonized with 1.5 or 15 μg/ml of HIV**^+^** IgG (Fig. 2). Killing was Env specific since control uncoated CEM cells in the same well were not killed in the presence of 1.5 or 15 μg/ml of HIV**^+^** IgG (Fig. 1A and 2A and B). However, varying the source of PBMC effector cells led to differences in the frequency of AnV^+^ target cells generated in this assay (Fig. 2A and B). These differences were attributable to the proportions of NK cells among the PBMC effector cells, as the frequency of NK cells among PBMCs correlated positively with the frequency of the AnV^+^ target cells generated in the ADCC-AnV assay (*P* = 0.021 and *r* = 0.766 for 1.5 μg/ml of HIV**^+^** IgG and *P* = 0.014 and *r* = 0.8 for 15 μg/ml of HIV**^+^** IgG) (Fig. 2C and D). Furthermore, the mean fluorescence intensity (MFI) of CD16 on the NK cells among the PBMC effector cells showed an even stronger correlation with the frequency of AnV ^+^ target cells generated in this assay, demonstrating the crucial role of CD16 in ADCC activity (*P* = 0.004 and *r* = 0.87 for 1.5 μg/ml of HIV**^+^** IgG and *P* = 0.003 and *r* = 0.88 for 15 μg/ml of HIV**^+^** IgG) (Fig. 2E and F). Next, we showed that negatively selected purified NK cells were superior to PBMCs at generating AnV^+^ target cells on a per-cell basis, whereas ADCC activity was almost completely abrogated when PBMCs were depleted of NK cells (see Fig. S1 in the supplemental material). Thus, among PBMCs, NK cells represented the main ADCC effector cell type under our experimental conditions.

10.1128/mBio.02690-19.1 FIG S1 NK cells are the major mediators of ADCC activity among PBMCs. cCEM cells labeled with CFSE were opsonized with 15 μg/ml (gray bars) and 150 μg/ml (black bars) of HIV^+^ IgG (filled bars) or HIV^−^ IgG (empty bars) and used as target (T) cells in the ADCC-AnV assay with the following effector (E) cells: PBMCs at an E:T ratio of 30:1, isolated NK cells at E:T ratios of 10:1 and 1:1, and NK cell-depleted PBMCs at E:T ratios of 10:1 and 1:1. NK cells were isolated by negative selection using magnetic beads, and the cells captured by from the beads were used as NK cell-depleted PBMCs. The *y*-axis data show percent ADCC as measured by the frequency of AnV^+^ cCEM cells after background subtraction. Error bars indicate standard deviations (SD) of results from replicates, and significance was determined by comparing the frequencies of AnV^+^ cCEM cells (% ADCC) between HIV^+^ IgG and HIV^−^ IgG for both concentrations (***, *P* < 0.001; 2-way ANOVA with Tukey’s multiple-comparison tests). Download FIG S1, EPS file, 0.4 MB. Copyright © 2019 Dupuy et al. 2019 Dupuy et al. This content is distributed under the terms of the Creative Commons Attribution 4.0 International license.

The ADCC-GranToxiLux (ADCC-GTL) assay measures the delivery of granzyme B (GzB) to target cells by flow cytometry (24). The ADCC-GTL assay indirectly measures apoptosis by measuring GzB activity, as GzB represents an early step in the cascade leading to target cell lysis by apoptosis (36). Therefore, we compared the ADCC-AnV and ADCC-GTL assays using isolated NK cells and increasing concentrations of HIV**^+^** IgG or of HIV^−^ IgG as a negative control (Fig. S2). While the results generated using the two assays were found to correlate, ADCC-AnV was significantly more sensitive at quantifying ADCC than ADCC-GTL in terms of the maximum frequency of apoptotic cells detected (*P* < 0.05 for the comparisons between AnV**^+^** and GzB**^+^** results for all HIV**^+^** IgG concentrations lower than 100 μg/ml) and of the superior signal/noise ratio achievable in the presence of HIV**^+^** IgG versus HIV^−^ IgG, particularly at low opsonizing Ab concentrations. Furthermore, the frequencies of ADCC-AnV**^+^** and ADCC-GzB**^+^** target cells peaked at 4 and 40 μg/ml of HIV**^+^** IgG, respectively (Fig. S2).

10.1128/mBio.02690-19.2 FIG S2 Measurement of ADCC activity using ADCC-AnV or ADCC-GTL assays with HIV^+^ IgG-opsonized cCEM cells. CFSE^+^ or NFL1^−^ TFL4^+^ labeled cCEM target cells (NFL1^−^ marks viable cells, and TFL1 marks target cells) were opsonized with increasing doses of HIV^+^ IgG (filled symbols) or HIV^−^ IgG (empty symbols) Ab and used as target cells in ADCC-AnV assays or in ADCC-GTL assays. The *y*-axis data show percent ADCC as measured by the frequency of annexin V^+^ (AnV; black symbols) or granzyme B^+^ (GzB; gray symbols) cCEM cells generated in the two ADCC assays after background subtraction. Error bars indicate SD of results from replicates, and significance was determined by comparing the percentages of ADCC between HIV^+^ IgG and HIV^−^ IgG for all IgG concentrations in each assay (**, *P* < 0.01; ***, *P* < 0.001) and by comparing the percentages of ADCC between AnV^+^ and GzB^+^ cells for all HIV^+^ IgG concentrations (##, *P* < 0.01; ###, *P* < 0.001). 2-way ANOVAs with Tukey’s multiple-comparison tests were used to assess the significance of between-group differences. Download FIG S2, EPS file, 0.3 MB. Copyright © 2019 Dupuy et al. 2019 Dupuy et al. This content is distributed under the terms of the Creative Commons Attribution 4.0 International license.

### Binding characteristics and ADCC competence of a panel of anti-HIV Envelope (Env)-specific MAbs with respect to newly infected CEM cells and bystander CEM cells.

The rgp120 commonly used to coat target cells for ADCC assays is monomeric and does not expose the same epitopes as native, trimeric Env on cells infected with replication-competent HIV (37). Quaternary epitopes on trimeric Env are not present on monomeric gp120. In contrast, monomeric gp120 displays the epitopes which are normally occluded inside the closed conformation of Env on target cells infected with wild-type HIV (16). Moreover, the CD4 binding site (CD4bs) Env epitope is not exposed on gp120-coated cells due to the engagement of the gp120 CD4bs with cell surface CD4 (16). This prompted us to develop a model of ADCC using HIV-infected instead of rgp120-coated cells. The virus used to infect CEM cells expressed all viral proteins from an NL4-3 backbone with the exception of Env, which came from HIV-Bal, and the heat shock protein heat-stable stable antigen (HSA) reporter gene (38). Since only a fraction of these cells were found to be HIV infected at 4 days postinfection, HSA staining was used to distinguish newly infected CEM cells from uninfected bystander CEM cells. This staining allowed us not only to gate on infected cells but also to evaluate binding and ADCC activity mediated by anti-Env Abs on newly infected CEM cells compared to uninfected bystander CEM cells whose CD4 had bound shed gp120, an interaction that revealed gp120-CD4i epitopes not exposed on newly infected CEM cells (16, 17).

**FIG 3:**
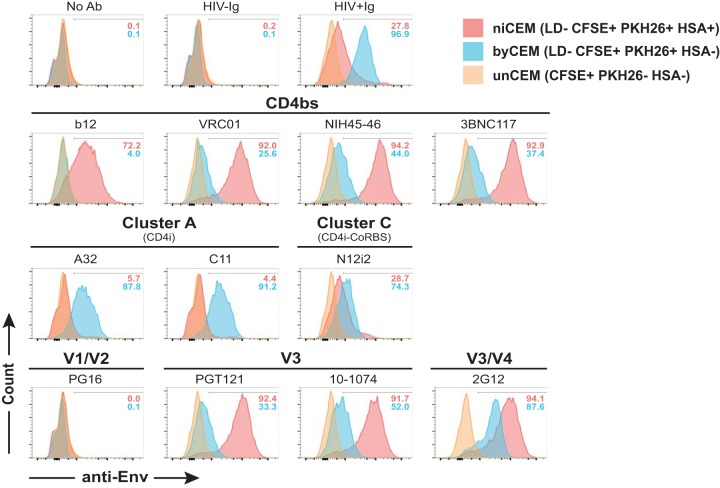
Binding of anti-Env-specific monoclonal and polyclonal antibodies (Abs) to newly infected CEM (niCEM), bystander CEM (byCEM), and uninfected CEM (unCEM) cells. CEM cells infected with the NL4-3–Bal–IRES–HSA virus were labeled 4 days postinfection (p.i.) with CFSE, LD (to eliminate cells that had died prior to the start of the ADCC assay), and PKH26 and were combined with unCEM cells labeled with CFSE only. The combined CEM cells were opsonized with 15 μg/ml of each anti-Env Ab identified above each histogram and then stained with anti-human IgG Fc-specific secondary Ab to detect anti-Env binding and with anti-HSA to differentiate niCEM (LD^−^ CFSE^+^ PKH26^+^ HSA^+^, in red) from byCEM (LD^−^ CFSE^+^ PKH26^+^ HSA^−^, in blue) and unCEM cells (CFSE^+^ PKH26^−^ HSA^−^, in orange). Each histogram depicts the properties of binding of each Ab in the panel to the three target cells (see also Fig. S3). Frequencies of Env^+^ niCEM cells and byCEM cells are indicated in each histogram. Data represent results from one experiment representative of three.

The binding characteristics of a panel of anti-Env MAbs and polyclonal HIV^+^ IgG (15 μg/ml each) to newly infected CEM cells (Live/Dead [LD]^−^ CFSE^+^ PKH26^+^ HSA^+^), bystander CEM cells (LD^−^ CFSE^+^ PKH26^+^ HSA^−^), and uninfected CEM cells (CFSE^+^ PKH26^−^) are shown in Fig. 3 (see also Fig. S3). As expected, control staining performed with no Ab and with HIV^−^ IgG generated similar levels of background staining of the three target cells. CD4bs Abs (b12, VRC01, NIH45-46, and 3BNC117) bound newly infected CEM cells with a much higher MFI than was seen with bystander CEM cells. This was expected since the CD4 binding site of gp120 is not available on bystander CEM cells by virtue of its interaction with cell surface CD4 (16). Abs to glycans such as PGT121, 10-1074, and 2G12 also recognized newly infected CEM cells better than bystander CEM cells. In contrast, Abs to CD4i epitopes such as A32, C11, and N12-i2 recognized bystander CEM cells with a higher MFI than newly infected CEM cells. Of note, polyclonal HIV**^+^** IgG stained both newly infected CEM cells and bystander CEM cells, with binding to bystander CEM cells resulting in a higher MFI.

10.1128/mBio.02690-19.3 FIG S3 Binding of anti-Env monoclonal and polyclonal Abs to niCEM cells, byCEM cells, and unCEM cells. CEM cells infected with the NL4-3–Bal–IRES–HSA virus were labeled 4 days postinfection (p.i.) with CFSE, LD, and PKH26 and combined with uninfected CEM (unCEM) cells labeled with CFSE only as described for Fig. 3. The combined CEM cells were opsonized with 15 μg/ml of each of the anti-Env Abs identified below each bar and were then stained with anti-human IgG Fc-specific secondary Ab to detect anti-Env binding and with anti-HSA to differentiate niCEM cells (LD^−^ CFSE^+^ PKH26^+^ HSA^+^; A and B, red bars) from byCEM cells (LD^−^ CFSE^+^ PKH26^+^ HSA^−^; C and D, blue bars) and unCEM cells (CFSE^+^ PKH26^−^ HSA^−^; E and F, orange bars). The frequency of Env^+^ cells (Env binding [%]; A, C, and E) and the intensity of Env binding (Env binding [MFI]; B, D, and F) are shown for each Ab in the panel for the three target cells. Data represent results from one experiment representative of three. Download FIG S3, EPS file, 2.3 MB. Copyright © 2019 Dupuy et al. 2019 Dupuy et al. This content is distributed under the terms of the Creative Commons Attribution 4.0 International license.

Overall, these observations suggested that gp120 was being shed from newly infected CEM cells and taken up by bystander CEM cells. They confirmed that CD4i epitopes are more readily exposed on bystander CEM cells and that CD4bs and V3 loop Abs targeting the closed Env conformation recognized newly infected CEM cells with a higher MFI than was seen with bystander CEM cells.

**FIG 4:**
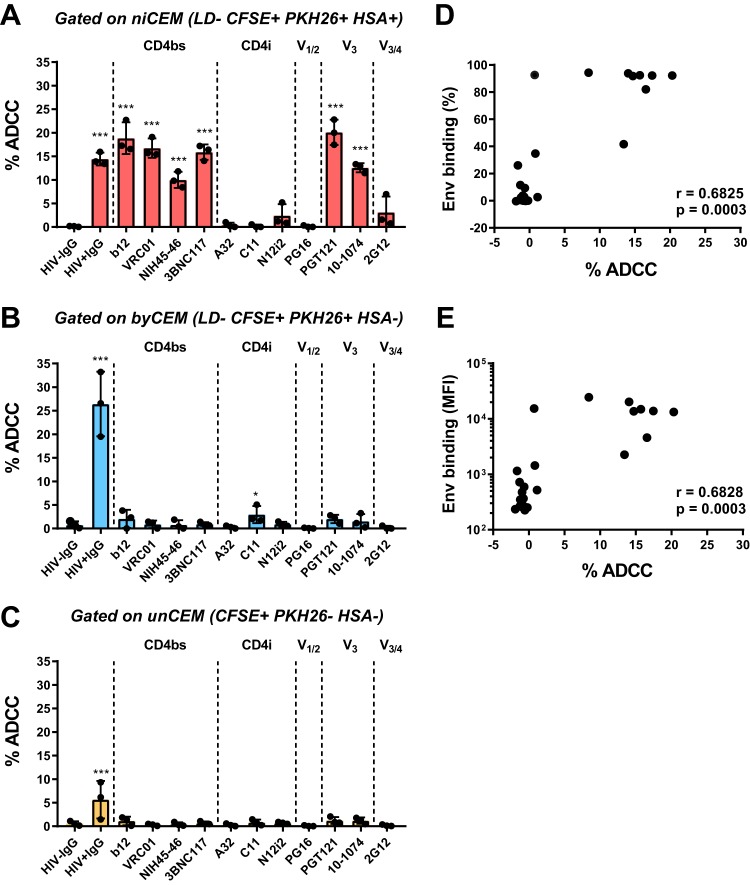
ADCC activity mediated by anti-Env monoclonal and polyclonal Abs to niCEM cells, byCEM cells, and unCEM cells (A to C) Four days p.i., infected CEM cells were labeled with CFSE, LD, and PKH26 and combined with uninfected CEM (unCEM) cells labeled with CFSE only as described for Fig. 3. The combined CEM cells were opsonized with 15 μg/ml of each anti-Env Abs used as described for Fig. 3 and incubated with effector NK cells for 1 h followed by staining with anti-HSA and AnV. ADCC activity (% ADCC) was measured as the frequency of AnV^+^ cells among niCEM (LD^−^ CFSE^+^ PKH26^+^ HSA^+^, red bars; A), byCEM (LD^−^ CFSE^+^ PKH26^+^ HSA^−^, blue bars; B) and unCEM (CFSE^+^ PKH26^−^, orange bars; C) cells. Data represent averages ± SD of results from three independent experiments. Each dot represents a single NK cell donor. Significance was determined by comparing the percentages of ADCC between each anti-Env Ab used and HIV^−^ IgG (*, *P* < 0.05; **, *P* < 0.01; ***, *P* < 0.001; 2-way ANOVA, Dunnett’s multiple-comparison test). (D and E) Spearman correlations between the frequency of anti-Env-specific Ab binding ([Env binding %]; D) and the MFI of anti-Env binding ([Env binding MFI]; E) to niCEM cells, as described for Fig. 3 (red histograms), with the percentage of ADCC generated using the same Abs to opsonize target cells.

Results of ADCC activity assays using the same panel of anti-Env MAbs and polyclonal HIV^+^ IgG (15 μg/ml each) to opsonize newly infected CEM cells, bystander CEM cells, and uninfected CEM cells are shown in Fig. 4A to C. All MAbs that bound newly infected CEM cells also supported ADCC measured as the frequency of AnV**^+^** target cells. The one exception to this was the gp120 outer domain recognizing 2G12 MAb, known to bind both the open and closed Env conformations but also to mediate poor ADCC activity (4, 15, 30). Indeed, 2G12 bound Env on both newly infected CEM cells and bystander CEM cells with a high MFI without triggering the ADCC of these target cells. 2G12 has an unusual domain-swapped configuration which may support 2G12 dimerization. This might affect the ability of this Ab to interact with Fc receptors to support ADCC (39, 40). The hypothesis regarding the association between binding and ADCC was supported by the significant correlation between the frequency and MFI of Ab binding and the frequency of AnV**^+^** newly infected CEM cells generated in the ADCC-Anv assay (*P* = 0.0003 and *r* = 0.6825 for percentages and *P* = 0.0003 and *r* = 0.6828 for MFI values, respectively) (Fig. 4D and E). In contrast to the results determined with newly infected CEM cells, none of the MAbs tested in the panel mediated robust ADCC of bystander CEM cells, with the exception of polyclonal HIV**^+^** IgG (Fig. 4B).

Gp120 shedding, like viral production, requires Env to be expressed on the surface of infected cells. In a mixture of infected and uninfected cells, uptake by bystander CEM cells of gp120 shed from newly infected CEM cells and infection of bystander CEM cells may occur simultaneously. Thus, to better characterize the infection status of bystander CEM cells in our system, we evaluated levels of intracellular p24 and cell surface CD4 in both HSA^+^ and HSA^−^ CEM cells 4 days postinfection. As shown in Fig. S4, HSA^+^ newly infected CEM cells expressed high levels of intracellular p24 and virtually no cell surface CD4, whereas HSA^−^ bystander CEM cells displayed cell surface CD4 and intracellular p24 at levels intermediate between those of newly infected CEM cells and uninfected CEM cells in these cultures. This phenotype may be related to the unexpectedly low binding observed with CD4bs Abs on bystander CEM cells (Fig. 3B) (see also Fig. S3C and D).

10.1128/mBio.02690-19.4 FIG S4 Cell surface CD4 and intracellular p24 expression levels in niCEM cells, byCEM cells, and uninfected CEM (unCEM) cells. CEM cells prepared as described for Fig. S3 were stained for intracellular p24 (top panel) and CD4 (bottom panel). Histograms show staining for p24 or CD4 in CFSE^+^ PKH26^+^ HSA^+^ niCEM cells (red histograms), CFSE^+^ PKH26^+^ HSA^−^ byCEM cells (blue histograms), and CFSE^+^ PKH26^−^ HSA^−^ unCEM cells (orange histograms). p24 and CD4 MFI data from the three target cells are indicated in each histogram. This experiment was repeated three times with similar results. Download FIG S4, EPS file, 1.1 MB. Copyright © 2019 Dupuy et al. 2019 Dupuy et al. This content is distributed under the terms of the Creative Commons Attribution 4.0 International license.

Nonetheless, levels of Env exposed on bystander CEM cells were insufficient to support high frequencies of AnV^+^ bystander CEM cells opsonized with any of the MAbs tested, though opsonization with HIV**^+^** IgG did support substantial ADCC as measured by the frequency of AnV^+^ bystander CEM target cells (Fig. 4B).

### Binding characteristics and ADCC competence of a panel of anti-Env-specific MAbs with respect to sorted HIV-infected CEM cells and coated CEM cells.

**FIG 5:**
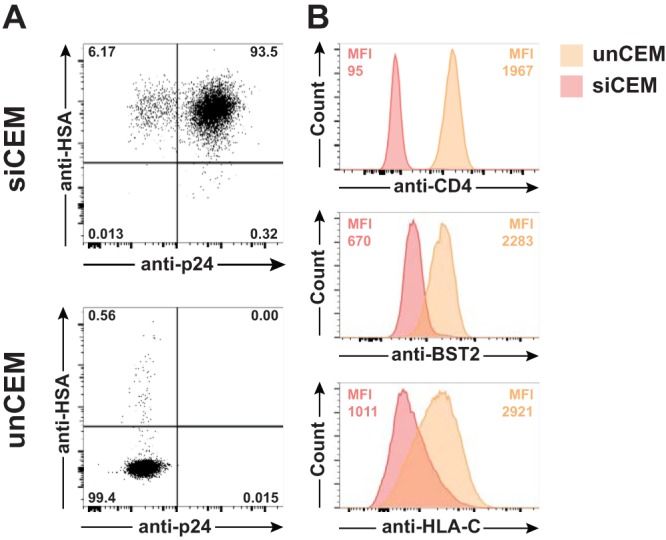
Direct and nondirect markers of infection expressed by sorted infected CEM (siCEM) cells. (A) Expression of cell surface HSA and intracellular p24 in siCEM cells (top) compared to uninfected cells (unCEM) (bottom). (B) Downregulation of cell surface CD4, BST-2, and HLA-C in siCEM cells (red) compared to unCEM cells (orange). The MFI levels determined for these markers in siCEM cells and unCEM cells are indicated in each histogram. The staining performed as described for panels A and B was repeated twice.

To rule out misinterpretations of these results due to the infection status of bystander CEM cells or the potential bias induced by the viral inoculum used, we enriched and sorted HSA^+^ newly infected CEM cells. These HIV-infected cells grow as an immortalized cell line and are referred to as sorted infected CEM cells here. The proportions of sorted infected CEM cells that expressed cell surface HSA and intracellular p24 were 99% and 94%, respectively (Fig. 5A). The downregulation of CD4, BST-2, and HLA-C from the surface of sorted infected CEM cells was consistent with these cells expressing functional Nef and Vpu (Fig. 5B).

**FIG 6:**
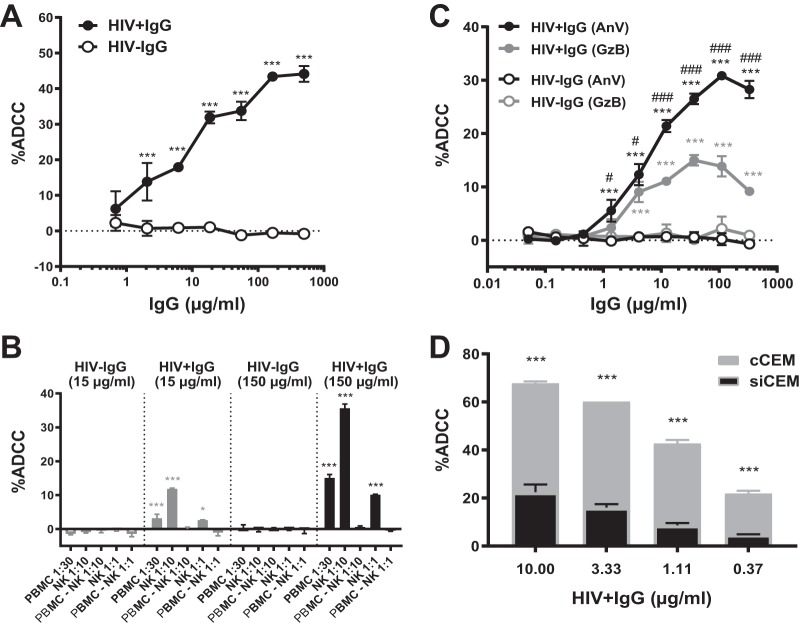
Characterization of the ADCC-AnV assay using HIV^+^ IgG Ab-opsonized siCEM cells as target cells. (A) siCEM target cells were labeled with CFSE, opsonized with increasing doses of HIV^+^ IgG (filled symbols) or HIV^−^ IgG (empty symbols), and used as target cells in an ADCC-AnV assay with NK cells as effector cells. Data represent averages ± SD of results from two donors of NK cells, and significance was determined by comparing the frequencies of AnV^+^ siCEM cells (%ADCC) between HIV^+^ IgG and HIV^−^ IgG for all IgG concentrations after background (No Ab) subtraction (***, *P* < 0.001). Data represent results from one experiment representative of three. (B) siCEM target (T) cells prepared as described for panel A were opsonized with 15 μg/ml (gray bars) and 150 μg/ml (black bars) of HIV^+^ IgG (filled bars) or HIV^−^ IgG (empty bars) and used as target cells in the ADCC-AnV assay with the following effector (E) cells: PBMCs at an E:T ratio of 30:1, isolated NK cells at E:T ratios of 10:1 and 1:1, and NK cell-depleted PBMCs at E:T ratios of 10:1 and 1:1. Error bars indicate SD of results from replicates, and significance was determined by comparing the frequencies of AnV^+^ siCEM cells (% ADCC) between HIV^+^ IgG and HIV^−^ IgG for both concentrations (*, *P* < 0.05; ***, *P* < 0.001). (C) CFSE^+^ or NFL1^−^ TFL4^+^ labeled siCEM T cells (NFL1^−^ marks viable cells and TFL1 marks target cells) were opsonized with increasing doses of HIV^+^ IgG (filled symbols) or HIV^−^ IgG (empty symbols) and used in an ADCC-AnV assay (black symbols) or in an ADCC-GTL assay (gray symbols). Error bars indicate SD of results from replicates, and significance was determined by comparing the percentages of ADCC as measured by the frequency of AnV^+^ or granzyme B^+^ (GzB^+^) siCEM between HIV^+^ IgG and HIV^−^ IgG for all IgG concentrations (***, *P* < 0.001) and by comparing the percentages of ADCC using AnV^+^ or GzB^+^ for all HIV^+^ IgG concentrations (#, *P* < 0.05; ###, *P* < 0.001). (D) siCEM target cells labeled with CFSE and PKH26 were combined 1:1 with cCEM cells labeled with CFSE before opsonization with HIV^+^ IgG and coculture with NK cells. Error bars indicate the SD of results from replicates, and significance was determined by comparing the percentages of ADCC as measured by the frequencies of AnV^+^ between siCEM cells (black bars) and cCEM cells (gray bars) for each opsonizing HIV^+^ IgG concentration (***, *P* < 0.001). Data represent results from one experiment representative of three. Two-way ANOVAs with Tukey’s multiple-comparison test were used for all comparisons in panels A, B, C, and D.

Results of ADCC-AnV assays performed using sorted infected CEM target cells opsonized by increasing doses of HIV**^+^** IgG and HIV^−^ IgG are shown in Fig. 6. Killing of sorted infected CEM cells by effector cells was dose dependent and anti-Env specific since HIV^−^ IgG did not mediate ADCC activity (Fig. 6A). The ADCC-AnV assay effector cells were NK cells as their depletion from PBMC abrogated the ADCC activity of sorted infected CEM cells, as previously described for coated CEM cells (Fig. 6B; see also Fig. S1). The results of ADCC-AnV and ADCC-GTL assays using sorted infected CEM cells as targets were correlated (Fig. 6C). As observed for coated CEM target cells, the ADCC-AnV assay was significantly more sensitive at quantifying ADCC activity than the ADCC-GTL assay in terms of the maximum frequency of apoptotic cells generated with HIV**^+^** IgG-opsonized sorted infected CEM cells (*P* < 0.05 for the comparisons between AnV**^+^** and GzB**^+^** for all HIV**^+^** IgG concentrations higher than 1 μg/ml) (Fig. 6C). Of note, the concentration of polyclonal HIV**^+^** IgG needed to obtain an equivalent frequency of AnV^+^ target cells in the ADCC-AnV assay was at least 10 times lower with coated CEM cells than with sorted infected CEM target cells (compare Fig. 6A, C, and D to Fig. 1B and Fig. S2). Staining of coated CEM cells and sorted infected CEM cells with increasing doses of HIV**^+^** IgG demonstrated that the superior killing of coated CEM cells with HIV**^+^** IgG in ADCC-AnV assays was consistent with the binding potential of HIV**^+^** IgG to these target cells (Fig. S5). Indeed, equivalent concentrations of HIV^+^ IgG bound a higher frequency of coated CEM cells with a higher MFI than sorted infected CEM cells (Fig. S5A and B). When MAb 2G12 was used instead of HIV**^+^** IgG to stain these target cells, the MFI of binding was higher on sorted infected CEM cells than on coated CEM cells (Fig. S5D). Therefore, the superior binding and ADCC activity characteristics of HIV^+^ IgG seen with coated CEM target cells were not due to the amount of Env exposed on coated CEM cells versus sorted infected CEM cells but rather suggested that a majority of the anti-Env Abs in polyclonal HIV^+^ IgG recognized epitopes exposed by monomeric/linear Env rather than the native trimeric, closed Env conformation.

10.1128/mBio.02690-19.5 FIG S5 Comparison of levels of HIV^+^ IgG and 2G12 mAb binding to Env on siCEM cells, cCEM cells, and uninfected CEM (unCEM) cells. siCEM cells and cCEM cells, separately labeled with CFSE and PKH26, were combined with unCEM cells labeled with CFSE only. The combined cells were opsonized with increasing concentrations of HIV^+^ IgG (A and B) or 2G12 mAb (C and D). Ab binding was detected using anti-human IgG Fc-specific secondary Ab. Anti-HSA staining distinguished siCEM cells (CFSE^+^ PKH26^+^ HSA^+^, red symbols) from cCEM cells (CFSE^+^ PKH26^+^ HSA^−^; blue symbols) and unCEM cells (CFSE^+^ PKH26^−^ HSA^−^; orange symbols). The *y*-axis data show the frequency of Env^+^ cells (Env binding [%]; A and C) and the intensity of Env binding (Env binding [MFI]; B and D) to each target cell. Download FIG S5, EPS file, 0.6 MB. Copyright © 2019 Dupuy et al. 2019 Dupuy et al. This content is distributed under the terms of the Creative Commons Attribution 4.0 International license.

**FIG 7:**
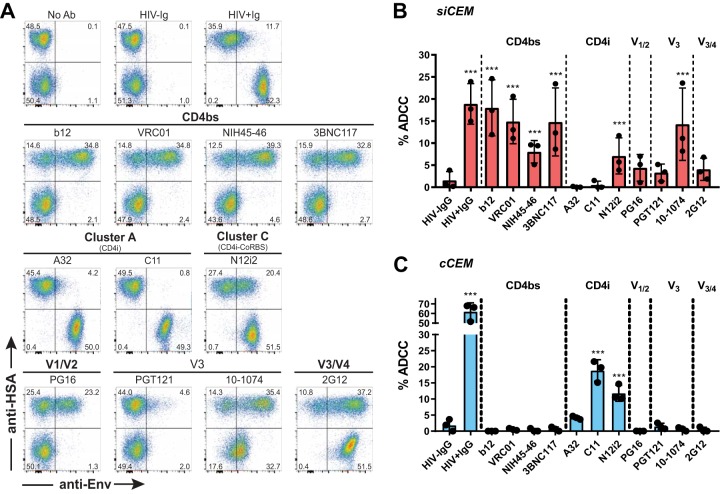
Binding to cCEM and siCEM and ADCC activity mediated by anti-Env monoclonal and polyclonal Abs. siCEM cells and cCEM target cells were separately labeled with CFSE, opsonized with 15 μg/ml of each anti-Env Abs used as described for Fig. 3. (A) Opsonized siCEM cells and cCEM cells were combined 1:1 and stained with anti-HSA (*y* axis) to differentiate siCEM cells (CFSE^+^ HSA^+^) from cCEM cells (CFSE^+^ HSA^−^), and anti-human IgG Fc-specific secondary Ab was used to detect anti-Env binding (*x* axis). The primary anti-Env Ab used for staining is identified above each density plot. Frequencies of HSA^+^ and/or Env^+^ cells are indicated in each quadrant. (B and C) Opsonized siCEM cells and cCEM cells were incubated side by side with isolated NK effector cells for 1 h. The *y* axes show ADCC activity (% ADCC) mediated by each of the anti-Env-specific MAbs (identified below each bar) measured as the frequencies of AnV^+^ siCEM cells (B) and cCEM cells (C). Data represent averages ± SD of results from three independent experiments. Each dot represents a single NK cell donor. Significance was determined by comparing the percentages of ADCC between the anti-Env Abs used with HIV^−^ IgG (*, *P* < 0.05; **, *P* < 0.01; ***, *P* < 0.001; 2-way ANOVA, Dunnett’s multiple-comparison test) (see also Fig. S6).

We next explored the characteristics of binding of the MAb panel to sorted infected CEM cells and coated CEM cells side by side and the frequency of AnV^+^ cells generated in the ADCC-AnV assay when target cells were opsonized using 15 μg/ml of each Ab in the panel (Fig. 7 and Fig. S6). Coated CEM cells were used here as a surrogate for the bystander CEM cells present in CEM cell cultures subjected to 4 days of HIV infection. As expected, the sorted infected CEM cells, like newly infected CEM cells, were preferentially recognized by CD4bs (b12, VRC01, NIH45-46, 3BNC117), 10-1074, and 2G12 MAbs (Fig. 3 and 7; see also Fig. S3A and B and S6). Of note, the PGT121 MAb poorly recognized sorted infected CEM cells compared to newly infected CEM cells. In contrast, coated CEM cells, like bystander CEM cells, were recognized by Abs to CD4i epitopes (A32, C11, and N12-i2) and 2G12, which binds to both “open” and “closed” conformations of Env (16). However, unlike the results seen with bystander CEM cells, we observed no binding of CD4bs MAbs or of PGT121 to coated CEM cells (Fig. 3 and 7; see also Fig. S3B and S6C and D). The pattern of Ab binding to sorted infected CEM cells and coated CEM cells correlated with their ability to support ADCC activity, and, with the exception of HIV**^+^** IgG, the global levels of ADCC using sorted infected CEM cells were higher than those seen using coated CEM cells as targets (Fig. 7B and C). Furthermore, when sorted infected CEM cells were used as ADCC-AnV target cells, the frequency and MFI of Env binding were positively correlated with the frequency of AnV^+^ target cells generated in this ADCC assay (Fig. S7). This was also the case when coated CEM cells were used as target cells, though the correlation was weaker (data not shown).

10.1128/mBio.02690-19.6 FIG S6 Binding of anti-Env monoclonal and polyclonal Ab to siCEM cells and cCEM cells. siCEM cells and cCEM target cells labeled with CFSE were combined and opsonized with 15 μg/ml of each of the anti-Env Abs indicated below each histogram as described for Fig. 7A. Ab binding was detected using anti-human IgG Fc-specific secondary Ab. Anti-HSA staining distinguished the siCEM cells (CFSE^+^ HSA^+^; A and B, red bars) from the cCEM cells (CFSE^+^ HSA^−^; C and D, blue bars). The *y*-axis data show the frequency of Env^+^ cells (Env binding [%]; A and C) and the intensity of Env binding (Env binding [MFI]; B and D) for each target cell. Data represent results from one experiment representative of three. Download FIG S6, EPS file, 1.2 MB. Copyright © 2019 Dupuy et al. 2019 Dupuy et al. This content is distributed under the terms of the Creative Commons Attribution 4.0 International license.

10.1128/mBio.02690-19.7 FIG S7 Correlation between anti-Env-specific monoclonal and polyclonal Ab binding to siCEM cells and the frequency of AnV^+^ cells generated in the ADCC-AnV assay. The *y*-axis data show the frequency (Env binding [%]; A) and MFI (Env binding [MFI]; B) of Env binding to siCEM cells by the use of each monoclonal and polyclonal Ab as described for Fig. 7. These values were correlated with the frequency of AnV^+^ siCEM cells generated in the ADCC-AnV (% ADCC) assay. Data represent results from one experiment representative of three. Download FIG S7, EPS file, 0.3 MB. Copyright © 2019 Dupuy et al. 2019 Dupuy et al. This content is distributed under the terms of the Creative Commons Attribution 4.0 International license.

### ADCC activity of plasma or IgG isolated from HIV^+^ subjects following blocking of CD4i epitopes with Fab fragments.

**FIG 8:**
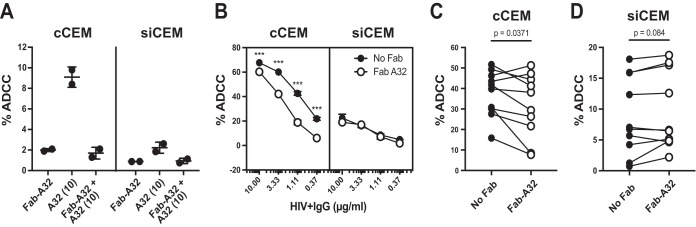
Inhibition of the ADCC-AnV activity of HIV^+^ IgG or HIV^+^ plasma samples using Fab fragments prepared from CD4i-specific MAb A32. cCEM cells and siCEM target cells were separately labeled with CFSE and preincubated with 10 μg/ml of A32 Fab or left untreated. cCEM cells and siCEM cells were then opsonized with A32 MAb or HIV^+^ IgG or HIV^+^ plasma samples and used as target cells in the ADCC-AnV assay. (A) Frequencies of AnV^+^ cells (% ADCC) among CFSE^+^ cCEM cells (left panel) and CFSE^+^ siCEM cells (right panel) induced by ADCC following opsonization with A32 Fab alone or 10 μg/ml of A32 MAb to target cells preincubated or not with A32 Fab fragments. Data represent averages ± SD of results from two NK cell donors. This experiment was repeated three times. (B) Frequencies of AnV^+^ results (% ADCC) in CFSE^+^ cCEM cells (left panel) and CFSE^+^ siCEM cells (right panel) induced by ADCC following opsonization of target cells by treatment with 0.37, 1.11, 3.33, and 10 μg/ml of HIV^+^ IgG preincubated with 10 μg/ml of A32 Fab or left untreated. Error bars indicate SD of results from replicates, and significance was determined by comparing the percentages of ADCC with and without Fab for each opsonizing HIV^+^ IgG concentration (***, *P* < 0.001; 2-way ANOVA with Tukey’s multiple-comparison tests). This experiment was repeated two times. (C and D) Frequencies of AnV^+^ (% ADCC) in CFSE^+^ cCEM cells (C) and CFSE^+^ siCEM cells (D) induced by ADCC following opsonization with 15 μg/ml of total IgG from 10 individual HIV^+^ plasma samples to target cells preincubated with A32 Fab or left untreated. Each point represents the average of results from duplicate assays for a single NK cell donor. Significance was determined by comparing the percentages of ADCC between no-Fab and A32 Fab pretreatment conditions (*P* values for these comparisons are shown in each panel (Wilcoxon tests).

**FIG 9:**
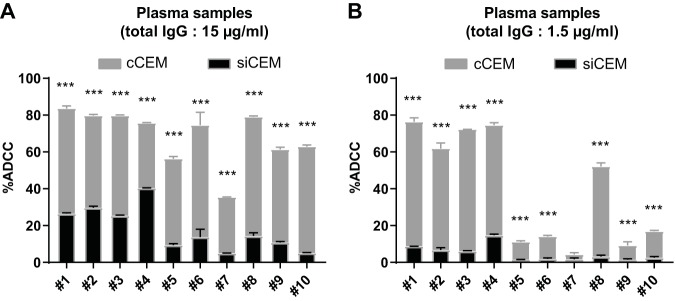
Anti-Env Abs in HIV^+^ plasma samples preferentially support ADCC of cCEM cells over siCEM cells. siCEM cells labeled with CFSE and PKH26 were combined 1:1 with cCEM cells labeled with CFSE only before opsonization with 10 individual HIV^+^ plasma samples and were cocultured with NK effector cells. The *y* axes show percent ADCC as measured by the superimposed frequencies of AnV^+^ siCEM cells (CFSE^+^ PKH26^+^; black histograms) and cCEM cells (CFSE^+^ PKH26^−^; gray histograms) with 15 μg/ml (A) and 1.5 μg/ml (B) of total IgG from each plasma sample used to opsonize target cells. Error bars indicate SD of results from replicates, and significance was determined by comparing the percentages of ADCC between siCEM cells and cCEM cells for each individual plasma sample (***, *P* < 0.001; 2-way ANOVA, Sidak’s multiple-comparison tests). Data represent results from one experiment representative of three.

Abs to cluster A-like epitopes, such as the prototypical A32 MAb, have been implicated as dominant ADCC-competent Abs in plasma from HIV-infected individuals. However, most of the work supporting this view used rgp120-coated target cells or target cells infected with HIV bearing mutant Nef and/or Vpu. Such HIV-infected cells retain cell surface CD4, which favors the assumption by Env of an open conformation (4, 15, 34). To evaluate the contribution of Abs with specificities that overlap those of A32 in HIV^+^ IgG and individual plasma samples from HIV^+^ subjects to the ADCC of coated CEM cells and sorted infected CEM cells, we pretreated these target cells with 10 μg/ml of A32 Fab fragment before opsonization with HIV^+^ IgG or HIV^+^ plasma (Fig. 8). As shown in Fig. 8A (see also Fig. S8), preincubation of target cells with 10 μg/ml of A32 Fab abolished the binding and ADCC competence of A32 MAb with respect to coated CEM cells. Pretreatment with the A32 Fab significantly reduced the ADCC activity mediated by HIV**^+^** IgG with respect to coated CEM cells. On average, there were 11%, 30%, 56%, and 72% decreases in the frequencies of AnV^+^ coated CEM cells generated when decreasing concentrations (i.e., 10, 3.3, 1.1, and 0.37 μg/ml) of HIV^+^ IgG were used to opsonize A32 Fab with pretreated coated CEM cells (*P* < 0.001 for comparisons between no Fab treatment and A32 Fab pretreatment for all HIV^+^ IgG concentrations; 2-way analysis of variance [ANOVA], Tukey’s multiple-comparison test). In contrast, pretreatment with the A32 Fab fragment failed to reduce the ability of HIV^+^ IgG to support ADCC-AnV activity on sorted infected CEM cells at any of the HIV^+^ IgG concentrations tested (Fig. 8B). These results suggest that HIV^+^ IgG contains anti-Env Abs with specificities that overlap those of A32 and support the idea of the ADCC activity of coated CEM cells. Furthermore, HIV^+^ IgG contains anti-Env Abs to epitopes other than A32 that are also capable of supporting ADCC of both coated CEM cells and sorted infected CEM cells (Fig. 8B). We next used individual plasma samples from 10 HIV-infected individuals to opsonize coated CEM cells (Fig. 8C) and sorted infected CEM cells (Fig. 8D). Pretreatment with the A32 Fab fragment alone was able to modestly but significantly reduce the ADCC competence of these HIV**^+^** plasma samples for coated CEM target cells. On average, A32 Fab pretreatment of coated CEM cells reduced ADCC activity by 16.2% (*P* = 0.037, Wilcoxon test) whereas A32 Fab pretreatment of sorted infected CEM cells had no effect on the frequency of AnV^+^ sorted infected CEM cells generated in the ADCC-AnV assay (Fig. 8C and D). This observation was consistent with sorted infected CEM cells expressing Env in a closed conformation unable to expose CD4i epitopes. In addition, by performing an ADCC-AnV assay with plasma from 10 HIV^+^ individuals on combined coated CEM cells and sorted infected CEM cells, we showed that anti-Env Abs in HIV^+^ plasma preferentially triggered the killing of coated CEM cells, as previously observed with HIV**^+^** IgG (Fig. 6D and Fig. 9). Indeed, the frequency of AnV^+^ target cells was between 3 to 7 times higher for coated CEM cells than for sorted infected CEM cells, depending on the concentration of HIV**^+^** IgG used for opsonization (Fig. 6D), and was on average between 4.5 and 8.2 times higher for coated CEM cells than for sorted infected CEM cells when 15 and 1.5 μg/ml of total IgG from the 10 HIV^+^ plasma samples were used, respectively (Fig. 9A and 9B).

10.1128/mBio.02690-19.8 FIG S8 CD4i-epitope-specific mAb A32 Fab fragments block binding of A32 mAb to cCEM but not to siCEM cells. siCEM cells and cCEM target cells labeled with CFSE were combined and preincubated with 10 μg/ml of A32 Fab or were left untreated before opsonization with 10 μg/ml of A32 mAb. Cells were then stained with anti-HSA to distinguish siCEM cells (CFSE^+^ HSA^+^) from cCEM cells (CFSE^+^ HSA^−^) by flow cytometry and with anti-human IgG Fc-specific secondary Ab to detect anti-Env binding. Histograms show overlaid staining generated binding for CFSE^+^ HSA^+^ siCEM cells (red histograms) and CFSE^+^HSA^−^ cCEM cells (blue histograms) with A32 Fab alone (top panel), A32 mAb alone (middle panel), and A32 Fab followed by A32 mAb (bottom panel) (anti-Env Abs). Frequencies of Env^+^ siCEM cells and cCEM cells are indicated in each histogram. Download FIG S8, EPS file, 1.2 MB. Copyright © 2019 Dupuy et al. 2019 Dupuy et al. This content is distributed under the terms of the Creative Commons Attribution 4.0 International license.

## DISCUSSION

We describe here a new method for ADCC quantification that measures the frequency of AnV^+^ cells as a readout for dead and dying cells. Using this assay and a panel of anti-Env MAbs to opsonize target coated CEM cells, newly infected CEM cells, bystander CEM cells, and sorted infected CEM cells, we confirmed that Abs to cluster A epitopes such as A32 and C11 predominantly recognized coated CEM cells and bystander CEM cells and supported killing of both types of target cells through ADCC. In contrast, CD4bs and V1/V2/V3 loop-specific Abs known to bind to the closed Env conformation (41) predominantly recognized newly infected CEM cells and sorted infected CEM cells, efficiently supporting their killing through ADCC. Env-specific Abs present in HIV^+^ plasma samples or HIV^+^ IgG supported the killing of all targets tested through ADCC, although a majority of this ADCC activity was directed toward epitopes found on the open Env conformation and exposed on coated/bystander CEM cells. A subset of the Env-specific Abs present in HIV^+^ IgG and HIV^+^ plasma recognized the Env closed conformation present on newly infected CEM cells and sorted infected CEM cells. Blocking experiments confirmed that the Ab specificities that overlapped A32-like epitopes in HIV^+^ plasma or HIV^+^ IgG supported the ADCC of coated CEM cells but not that of sorted infected CEM cells (see model in Fig. S9 in the supplemental material).

10.1128/mBio.02690-19.9 FIG S9 Model of ADCC using HIV-infected target cells exposing Env in a native, closed, trimeric conformation or Env in an open conformation. In HIV-infected cells (left panel), expression of Nef and Vpu downregulates CD4, which prevents its interaction with gp120, allowing Env to be exposed in its native closed trimeric conformation. Due to its inherent instability, gp120 sheds from infected cells and binds to CD4 on uninfected bystander cells (right panel), exposing Env in a CD4-bound conformation. Different categories of Env-specific Abs isolated from HIV-infected subjects bind to epitopes specifically exposed by either the native closed trimeric Env conformation (such as CD4bs Abs) or the CD4-bound conformation (such as CD4i Abs) or both conformations (such as 2G12). The recognition of the Fc portions of these Abs by CD16 on NK cells triggers the release of cytotoxic granules containing granzyme B and perforin, leading to the killing of target cells. In this work, we developed a new ADCC assay which employs annexin V to detect dying and dead cells following ADCC. This assay allowed us to measure the proportion of Env-specific ADCC-competent Abs in HIV-infected plasma, based on their capacity to recognize and eliminate infected, bystander, or Env rgp120-coated cells as a surrogate for shed gp120 on bystander cells. Using this assay, we showed that HIV^+^ plasma contains Env-specific Abs capable of recognizing and supporting ADCC of infected cells, though a majority were directed towards CD4i epitopes on bystander cells, a phenomenon that could possibly be associated with the CD4 depletion seen *in vivo*. Download FIG S9, EPS file, 1.5 MB. Copyright © 2019 Dupuy et al. 2019 Dupuy et al. This content is distributed under the terms of the Creative Commons Attribution 4.0 International license.

The ADCC-AnV assay that we describe here is easy to perform, high throughput, specific for target cells expressing HIV Env, and more sensitive than the ADCC-GTL assay. Unlike the widely used rapid fluorometric ADCC (RFADCC) assay, which quantifies membrane exchange between target and effector cells rather than ADCC activity, the ADCC-AnV assay measures ADCC activity by identifying and quantifying apoptotic target cells (42, 43). Staining for AnV to measure ADCC activity is rapid, as AnV^+^ target cells are detected after a 1-h incubation with effector cells. NK cells were confirmed to be the main ADCC-competent effector cell in the assay, and the MFI of CD16 expression on NK cells was directly associated with the ADCC-AnV assay readout. This assay was used to measure ADCC activity of Env-specific BnAbs and NnAbs MAb, HIV^+^ IgG, and plasma from individual HIV^+^ subjects by the use of target CEM cells either coated with rgp120 from HIV-Bal or infected with a virus expressing Env-Bal and HSA. Labeling target cells with CFSE/PKH26 or staining them with anti-HSA allowed us to specifically gate on infected cells (HSA^+^) and/or bystander cells (HSA^−^) exposed to shed gp120 by the use of flow cytometry and to include uncoated/uninfected CEM cells as within-well internal negative controls. Some investigators perform ADCC assays using an NK cell line expressing CD16 as a source of effector cells (30). The advantages of using an NK cell line are that the cells are readily available and are consistent from experiment to experiment. Employment of an NK cell line would eliminate the need for large blood draws (i.e., leukapheresis) to obtain enough NK cells from the same source for ADCC assays. It would also reduce the cost associated with isolation of NK cells from PBMCs. The use of an NK effector cell line would be worth exploring as a way to improve the ADCC assay described in this paper.

A panel of BnAbs and NnAbs to various HIV Env epitopes was used to assess their binding and ADCC competence. Anti-Env Abs with specificity to the CD4bs (i.e., b12, VRC01, NIH45-46, and 3BNC117) and glycan-dependent V3 loop (i.e., PGT121, 10-1074), but not to cluster A-specific MAbs, bound newly infected CEM cells. These results were consistent with wild-type Nef and Vpu expression in newly infected CEM downregulating cell surface CD4, which prevented the interaction with gp120 required to open the Env conformation that exposes CD4i epitopes. The Ab panel bound sorted infected CEM cells similarly to newly infected CEM cells with 2 exceptions. PG16, a V1/V2 loop-specific Ab recognizing a quaternary epitope on trimeric Env, bound only sorted infected CEM cells, whereas PGT121, a V3 loop-specific Ab recognizing the Env closed conformation, bound only newly infected CEM cells. In contrast, 10-1074, another V3 loop-specific Ab, bound both newly infected CEM cells and sorted infected CEM cells. For both newly infected CEM cells and sorted infected CEM target cells, the frequency and MFI of anti-Env binding were correlated with the frequency of AnV^+^ target cells generated in the ADCC-AnV assay.

Anti-cluster A-specific NnAbs A32 and C11 bound both bystander CEM cells and coated CEM cells but not newly infected CEM cells or sorted infected CEM cells. This finding is consistent with bystander and coated CEM target cells presenting gp120 in an open conformation, as has been reported previously by others (16, 17). Surprisingly, bystander CEM cells, unlike coated CEM cells, were also recognized, though weakly, by the CD4bs-specific BnAbs VRC01, NIH45-46, and 3BNC117. In HIV-infected cell cocultures, gp120 shed by infected cells binds bystander cells by virtue of its interaction with CD4 on these cells, preventing the binding of CD4bs-specific BnAbs (16, 33). Thus, the binding of CD4bs-specific BnAbs to bystander CEM is unlikely to be due to the recognition of shed gp120 from newly infected CEM cells. The low level of binding of CD4bs Abs to bystander CEM cells might have been due to recognition of defective viral particles attached to uninfected cells that originated from the surrounding newly infected CEM cells, which continuous produce HIV, or from the viral inoculum used to infect CEM cells. In line with this, Lee et al. suggested that the binding and ADCC activity observed with bystander CEM can be explained by the attachment of viral particles present in the inoculum, which has been shown to generate intermediate levels of p24 (33). Supporting this idea, our bystander CEM cells expressed insufficient HSA levels to be detectable as HIV^+^ by flow cytometry and p24 levels between those of newly infected CEM cells and uninfected CEM cells. Enough defective viral particles may be present to bind bystander CEM cells in a manner that exposes the CD4i epitopes that result from the formation of CD4-gp120 complexes while also maintaining gp120 trimers as previously suggested (33, 44). However, we cannot formally exclude the possibility that the bystander CEM cells were present at an early stage of the infection. In fact, we observed that incubation of uninfected CEM cells with supernatant from sorted infected CEM cells, which contains shed gp120 and viral particles, did not block the recognition of surface CD4 by detector OKT4 Ab (data not shown) and therefore cannot account for the partial CD4 downregulation seen on bystander CEM cells. Activity during an early stage of the infection might result in a partial downregulation of CD4 by the early expressed HIV Nef protein, as observed on HSA^−^ bystander cells, allowing some HIV Env to remain in a closed conformation recognized by CD4bs BnAbs and some HIV Env to expose CD4i epitopes due to interactions with CD4. Taking the data together, the challenges inherent in interpreting the anti-Env-specific BnAb and NnAb binding and ADCC competence results and the infection status of recently infected CEM cells provided the impetus to generate sorted infected CEM cells, which were virtually 100% HIV infected based on HSA, CD4, and p24 expression patterns. The availability of sorted infected CEM cells allowed us to compare these cells with coated CEM cells, which exposed CD4i epitopes in a manner similar to that seen with bystander cells in HIV-infected cocultures. Comparisons of sorted infected CEM cells and coated CEM target cells revealed that CD4bs-specific Abs exclusively recognized sorted infected CEM cells whereas the CD4i-specific NnAbs exclusively bound coated CEM cells.

ADCC-competent Abs in HIV^+^ plasma have been proposed to preferentially target the open Env conformation (15, 20). In line with this, Abs to cluster A determinants, such as A32, were described previously as dominant ADCC-competent Abs in HIV^+^ plasma (20, 25). However, this observation was made using target cells coated with rgp120 or infected with a virus unable to downregulate CD4, each of which exposes Env in an open conformation. To challenge this, we compared the capacities of polyclonal HIV^+^ IgG and individual HIV^+^ plasma to trigger ADCC of coated CEM cells and sorted infected CEM side by side, assuming that coated CEM cells can be used to measure levels of ADCC-competent Abs targeting an open Env/CD4i conformation and that sorted infected CEM cells can be used to measure levels of ADCC-competent Abs targeting a closed/trimeric Env conformation. We showed that polyclonal HIV^+^ IgG and HIV^+^ plasma elicited ADCC responses to both coated CEM cells and sorted infected CEM cells. The levels of ADCC were on average 6 times higher for coated CEM cells than for sorted infected CEM target cells opsonized with HIV^+^ plasma. This confirmed that the monomeric gp120/CD4i conformation and, by extension, gp120 shed by infected cells are preferentially targeted by anti-Env Abs in HIV^+^ plasma. This is despite the results seen with MAb 2G12, which detects a conformation-independent Env epitope, staining sorted infected CEM cells with a higher MFI than coated CEM cells. Since the open Env conformation preferentially marks bystander cells, these HIV^+^ plasma Abs may contribute to the killing of uninfected cells rather than to controlling HIV infection. Their role in HIV control can now be examined by using sorted infected CEM cells as ADCC target cells.

We investigated the contribution of A32-like Abs to ADCC responses. A32 Fab fragments partially blocked ADCC mediated by HIV^+^ IgG- and HIV^+^ plasma-opsonized coated CEM cells but failed to block ADCC mediated by HIV^+^ Ig-opsonized sorted infected CEM cells. As expected, the reduction in ADCC activity was restricted to coated CEM cells exposing CD4i epitopes. This observation suggested that the level of A32-like Abs mediating ADCC activity in HIV^+^ plasma was relatively modest and that they were strictly directed toward bystander CEM cells. These results highlight the presence of ADCC-competent Abs in HIV^+^ plasma to epitopes other than those recognized by A32 MAbs, capable of supporting the efficient killing of bystander cells displaying an open Env conformation and, to a lesser extent, of infected cells displaying a closed Env conformation. It would be of great interest to evaluate the relevance of bystander cell killing through the ADCC in the global CD4 depletion which occurs in HIV-infected donors since it is well known that only a small fraction of CD4^+^ cells are actually infected *in vivo* whereas the majority of apoptotic CD4^+^ cells in the lymph nodes of HIV^+^ persons consist of bystander CD4^+^ cells surrounding infected cells (17).

We envision that the ADCC-AnV assay described here using sorted infected CEM cells as target cells may be useful for immune monitoring of HIV vaccine trials and therapeutic approaches that aim to induce anti-Env-specific Abs. The ADCC-AnV assay would aid in distinguishing Abs with specificities directed at bystander cells, which may contribute to CD4 loss versus Abs able to recognize HIV-infected cells that support HIV control. The concept that Abs able to recognize HIV-infected cells can support their lysis through ADCC may have applications in the context of other viral infections. For example, both respiratory syncytial virus (RSV) and Ebola virus (EboV) encode forms of their viral glycoproteins that are secreted or shed from the infected cell surface such as occurs for HIV-infected cells (45–49). This phenomenon protects virus-infected cells. Anti-virus Abs bind the soluble glycoproteins, making them unavailable to bind infected cells. Strategies aimed at preventing shedding or at identifying epitopes maintained on virus-infected cells have the potential to improve Ab targeting of virally infected cells able to support ADCC.

## MATERIALS AND METHODS

### Ethics statement.

This study was conducted in accordance with the principles expressed in the Declaration of Helsinki. It was approved by the Institutional Review Boards of the Comité d’Éthique de la Recherche du Centre Hospitalier de l’Université de Montréal (17-096) and the Research Ethics Committee of the McGill University Health Centre (2018-4505). All individuals provided written informed consent for the collection of samples and subsequent analyses.

### Cells and reagents.

PBMCs used as effector cells in ADCC assays were obtained from HIV-uninfected subjects enrolled in the St Luc cohort of injection drug users or from a cohort of couples with discordant HIV characteristics. None of the study subjects met the criteria for consideration as HIV-exposed seronegative (HESN) subjects. PBMCs were isolated from leukapheresis samples by density gradient centrifugation, as previously described (50, 51). Cells were frozen in 90% fetal bovine serum (FBS; Wisent BioProducts, St-Jean-Baptiste, QC, Canada)–10% dimethyl sulfoxide (Sigma-Aldrich, St. Louis, MO) and stored in liquid nitrogen until use. Thawed PBMCs were rested overnight in RPMI 1640 medium supplemented with 10% FBS, 2 mM l-glutamine, 50 IU/ml penicillin, and 50 mg/ml streptomycin (R10; all from Wisent) before use.

CEM cells were obtained from the NIH AIDS Reagent Program, Division of AIDS (DAIDS), NIAID, NIH, as CEM.NKR.CCR5 cells (from Alexandra Trkola) (26, 27, 52). HIV-1 Bal rgp120 was obtained through the NIH AIDS Reagent Program (DAIDS, NIAID, NIH). Anti-HIV immune globulin (HIVIG; referred to here as HIV^+^ IgG), representing a pool of purified IgG from asymptomatic HIV-positive donors with CD4^+^ counts above 400/μl, was obtained from the National Agri-Food Biotechnology Institute (NABI) and the National Heart, Lung, and Blood Institute (NHLBI) through the NIH AIDS Reagent Program (DAIDS, NIAID, NIH) (53). Plasma from five healthy donors at low risk for HIV infection (referred to here as HIV^−^ IgG) was obtained from blood draws and stored in acid citrate dextrose-containing vacutainers. The tubes were centrifuged, the liquid phase was pooled, and total IgG was quantified by enzyme-linked immunosorbent assay (ELISA). A Live/Dead fixable dead cell stain kit (Invitrogen, St Laurent, QC, Canada) was used to quantify dead cells by flow cytometry. For some experiments, plasma was obtained from HIV-infected individuals enrolled in the Montreal Primary Infection Cohort or the Canadian Cohort of HIV-infected Slow Progressors.

### HIV infection of CEM target cells.

HIV-infected CEM cells were generated by infecting CEM cells with a replication-competent NL4.3-based HIV-1 virus expressing all viral genes from the original NL4.3 backbone except the Env gene, which was replaced by a Bal Env gene and a reporter gene encoding heat-stable antigen (HSA, murine CD24) coexpressed with Nef by the use of an internal ribosome entry site (IRES) sequence. The NL4-3–Bal–IRES–HSA viral construct was a kind gift from Michel Tremblay (Laval University, Quebec, QC, Canada) (38). CEM cells were infected with HIV by adding supernatant from 293T cells cotransfected with NL4-3–Bal–IRES–HSA and vesicular stomatitis virus glycoprotein G (VSV-G) plasmids to 10^6^ CEM cells by spinoculation at 2,000 × *g* for 90 min and incubating these cells for 30 min at 37°C in a 5% CO_2_ humidified incubator. After washing, the CEM cells were cultured in R10. Four days after infection, the CEM cells were on average 52% HSA^+^ (range, 45% to 73%).

In order to prepare infected CEM cells exclusively exposing Env in a closed conformation, newly infected CEM cells were stained with PECy7-conjugated anti-mouse CD24 Ab (BD Biosciences, Mississauga, ON, Canada), sorted for HSA expression using a FACSAria instrument (BD Biosciences), and expanded *in vitro*. The frequencies of CD4^+^, HLA-C^+^, and BST-2^+^ CEM cells were evaluated by surface staining with MAbs specific for CD4 (clone OKT4; BD Biosciences), HLA-C (clone DT-9; Biolegend, Burlington, ON, Canada), and BST-2 (NIH AIDS Reagent Program, DAIDS, NIAID, NIH), respectively, whereas the frequency of p24^+^ CEM cells was evaluated by intracellular staining using a phycoerythrin (PE)-conjugated anti-p24 antibody (clone KC57; Beckman-Coulter, Mississauga, ON, Canada). Sorted infected CEM cells expressed levels of ligands for NKG2D that were no higher than those seen with uninfected CEM cells (unpublished results) (54).

### IgG ELISA.

To detect the total amount of IgG in HIV^−^ and HIV^+^ plasma samples, we used a human IgG ELISA quantitation set (Bethyl Laboratories, Montgomery, TX) per the manufacturer’s instructions.

### Target cell labeling.

All target cells were stained with the green fluorescent cytosolic cell dye carboxyfluorescein succinimidyl ester (CFSE; Thermo Fisher Scientific, St. Laurent, QC, Canada) to distinguish them from PBMCs or NK effector cells. CFSE staining was performed per the manufacturer’s instructions and as previously described (55).

For binding experiments and ADCC assays using two target cells combined (i.e., rgp120-coated CEM cells or infected CEM cells 4 days postinfection (p.i.) with HIV combined with uncoated or uninfected CEM cells), CFSE^+^ cells were also stained with PKH26 red fluorescent membrane cell dye (PKH26 red fluorescent cell linker kit; Sigma-Aldrich) to distinguish them from CFSE^+^ PKH26^−^ uncoated or uninfected CEM control cells, used as an internal control for nonspecific binding and killing. PKH26 staining was performed as previously described (55).

### Preparation of rgp120-coated CEM target cells.

Labeled CEM cells were resuspended to reach a level of 1 × 10^6^ cells in 100 μl of R10 to which was added 0.5 μg of rgp120 (from the NIH AIDS Reagent Program) for 1 h at 37°C in a humidified 5% CO_2_ incubator. Excess rgp120 was washed off with R10.

### Preparation of effector cells.

PBMCs or NK cells were used as ADCC effector cells. Cryopreserved, thawed PBMCs were resuspended to a level of 2 × 10^6^ cells per ml of R10 and rested overnight in a 37°C, humidified 5% CO_2_ incubator. For the experiments performed with NK cells, the cells were enriched from PBMCs using a negative selection kit (EasySep human NK cell enrichment kit; STEMCELL, Vancouver, BC, Canada) per the manufacturer’s instructions. This kit does not include antibodies to CD16 or FcR blocking Abs, either of which could have an impact on ADCC assays. NK cell purity (average, 93% ± 7.2% CD56^+^ cells) and CD16 expression were evaluated by surface staining with MAbs specific for CD56 (clone HCD56; Biolegend), CD16 (clone 3G8; Biolegend) and CD3 (OKT3; Biolegend). Ninety-nine percent of CD56^dim^ CD3^−^ cells and 64% of CD56^bright^ CD3^−^ cells were CD16^+^ (see Fig. S10 in the supplemental material).

10.1128/mBio.02690-19.10 FIG S10 Purity and CD16 expression of NK cells isolated by negative selection. CD3^−^ CD56^+^ cells were gated on live PBMCs (top panel) and isolated NK cells (bottom panel). The frequencies of CD16 on CD3^−^ CD56^+^ NK cells (upper) and non-NK cells (lower) are shown in the top and bottom right-hand panels. Download FIG S10, EPS file, 1.8 MB. Copyright © 2019 Dupuy et al. 2019 Dupuy et al. This content is distributed under the terms of the Creative Commons Attribution 4.0 International license.

### ADCC-AnV assays.

For ADCC assays using coated CEM target cells, cells were stained with CFSE and PKH26 before being coated with rgp120. A total of 10^4^ CFSE^+^ PKH26^+^ coated CEM cells combined with 10^4^ CFSE^+^ PKH26^−^ uncoated CEM cells were plated into the wells of a 96-well V-bottom plate in 50 μl of R10. Target cells were opsonized by adding 50 μl of predetermined concentrations of Abs (HIV^+^ IgG, HIV^−^ IgG, BnAbs, NnAbs, or HIV^+^ plasma) for 20 min at room temperature (RT) in the dark. After Ab incubation, 100 μl of PBMC effector (E) cells were added to each well containing opsonized coated CEM target cells (T) at an E:T ratio of 30:1. Plates were centrifuged at 300 × *g* for 1 min to pellet the cells and incubated at 37°C in a humidified 5% CO_2_ incubator for 1 h.

For ADCC assays using infected CEM cells 4 days p.i., cells were stained with PKH26, CFSE, and Live/Dead stain such that HIV-infected cells that died prior to the start of the ADCC assay could be gated out. A total of 10^4^ CFSE^+^ PKH26^+^ infected CEM cells 4 days p.i. combined with 10^4^ CFSE^+^PKH26^−^ uninfected CEM cells were plated into the wells of a 96-well V-bottom plate followed by Ab opsonization. At 20 min after incubation with Abs, NK effector cells were added to each well containing target cells at an E:T ratio of between 5:1 and 10:1, unless otherwise specified. Plates were centrifuged and incubated as described above for 1 h. Cells were then stained with anti-HSA Ab to distinguish newly infected CEM cells, which were LD^−^ CFSE^+^ PKH26^+^ HSA^+^, from bystander CEM cells, which were LD^−^ CFSE^+^ PKH26^+^ HSA^−^.

For ADCC assays using sorted infected CEM target cells, 10^4^ CFSE^+^ sorted infected CEM cells were plated into the wells of a 96-well V-bottom plates and opsonized with Ab for 20 min. NK effector cells were then added to these wells at an E:T ratio of between 5:1 and 10:1, unless otherwise mentioned. Plates were centrifuged and incubated as described above for 1 h. In ADCC experiments comparing sorted infected CEM cells and coated CEM target cells in separate wells, both types of target cells were stained with CFSE and opsonized with Abs in parallel. In experiments where sorted infected CEM cells and coated CEM target cells were combined 1:1 and plated in the same well, they were distinguished by staining sorted infected CEM cells with PKH26 before opsonization or by staining with anti-HSA Ab after incubation with effector cells.

We used a new method to quantify ADCC activity in target CEM cells. This method employed AnV as a readout to identify and quantify the frequencies of both early and late apoptotic target cells following incubation of effector and Ab-opsonized target cells. The effector and opsonized target cells in each well were cocultured for 1 h, washed with 1× AnV binding buffer (BD Biosciences), and incubated with 100 μl of the same buffer supplemented with 1 μl of AnV (BD Biosciences) for 10 min at RT. Cells were washed and resuspended in 1× AnV binding buffer and acquired using an LSR Fortessa or LSR Fortessa X-20 instrument and a high-throughput system (HTS; BD Biosciences). Percentages of ADCC (% ADCC) were obtained by calculating the average frequency of AnV^+^ target cells from duplicate wells after subtracting the frequency of AnV^+^ cells measured under the no-Ab negative-control conditions. Results were analyzed using FlowJo software v10.

For some experiments, Fab fragments of the MAb A32 were used to pretreat coated CEM cells and sorted infected CEM target cells prior to Ab opsonization. Fab (10 μg/ml) was added to target cells, and incubation was performed for 20 min at RT before the addition of opsonizing Abs.

### ADCC-GranToXiLux (ADCC-GTL) assay.

The ADCC-GTL assays were performed as previously described (24, 56, 57).

### Ab panel used for ADCC and to characterize the Env structure on target cells.

The Env expressed on coated CEM cells, newly infected CEM cells, bystander CEM cells, and sorted infected CEM cells was probed using a panel of anti-Env-specific BnAbs and NnAbs, HIV^+^ IgG, and HIV^−^ IgG. These Abs targeted the following Env epitopes: CD4i anti-cluster A (A32 and C11), CD4i-coreceptor binding site (CoRBS) (N12-i2), CD4 binding site (CD4bs) (b12, VRCO1, NIH45-46 G54W, and 3BNC117), V3 glycan (PGT121), V3 loop (10-1074), the V1/V2 glycan (PG16), and the N-linked glycans on the gp120 outer domain (2G12). All these Abs were obtained from the NIH AIDS Reagent Program, with the exception of C11 and N12-i2. Table 1 lists these MAbs, their sources, and relevant references. The binding of unconjugated primary Abs was detected using an allophycocyanin (APC)-conjugated mouse anti-human IgG Fc-specific secondary MAb (BioLegend). Negative controls for staining included HIV^−^ IgG and staining with secondary Ab alone.

**TABLE 1:** Specificity and acknowledgments for anti-gp120 epitope-specific antibodies obtained from the NIH AIDS Reagent Program^*a*^

Antibody clone	Specificity	Source or reference(s)
b12	CD4bs	DAIDS, NIAID, NIH, from Dennis Burton and Carlos Barbas (58–61)
VRC01	CD4bs	DAIDS, NIAID, NIH, from John Mascola (62)
NIH45-46 G54W	CD4bs	DAIDS, NIAID, NIH, from Pamela Bjorkman (63)
3BNC117	CD4bs	DAIDS, NIAID, NIH, from Michel Nussenzweig (64)
A32	CD4i (cluster A)	DAIDS, NIAID, NIH, from James E. Robinson (28, 29)
C11	CD4i (cluster A)	18, 22, 65–67
N12-i2	CD4i-CoRBS (cluster C)	18
PG16	V1/V2 glycan	DAIDS, NIAID, NIH (68)
PGT121	V3 glycan	DAIDS, NIAID, NIH (69)
10-1074	V3	DAIDS, NIAID, NIH, from Michel Nussenzweig (64)
2G12	Glycan	DAIDS, NIAID, NIH, from Polymun Scientific (70–73)
HIV^+^ IgG	Polyclonal	DAIDS, NIAID, NIH; anti-HIV Immune Globulin (HIVIG) from NABI and NHLBI (53)

a CD4i, CD4-induced epitope; DAIDS, Division of AIDS; NIAID, National Institute of Allergy and Infectious Diseases; NIH, National Institutes of Health; CD4bs, CD4 binding site epitope; NABI, National Agri-Food Biotechnology Institute; NHLBI, National Heart, Lung, and Blood Institute.

### Statistical analysis.

Statistical analyses were conducted using GraphPad Prism 7.0 (La Jolla, CA, USA). Comparisons were conducted using nonparametric Wilcoxon tests to assess the significance of the differences between the two conditions and 2-way ANOVA tests with Tukey’s, Sidak’s, or Dunnett’s correction for multiple variables. Correlation analyses were performed using a nonparametric Spearman test. An α level of 5% was used for statistical significance.

## References

[1] Rerks-Ngarm S, Pitisuttithum P, Nitayaphan S, Kaewkungwal J, Chiu J, Paris R, Premsri N, Namwat C, de Souza M, Adams E, Benenson M, Gurunathan S, Tartaglia J, McNeil JG, Francis DP, Stablein D, Birx DL, Chunsuttiwat S, Khamboonruang C, Thongcharoen P, Robb ML, Michael NL, Kunasol P, Kim JH; MOPH-TAVEG Investigators. 2009 Vaccination with ALVAC and AIDSVAX to prevent HIV-1 infection in Thailand. N Engl J Med 361:2209–2220. doi:10.1056/NEJMoa0908492. 19843557

[2] Haynes BF, Gilbert PB, McElrath MJ, Zolla-Pazner S, Tomaras GD, Alam SM, Evans DT, Montefiori DC, Karnasuta C, Sutthent R, Liao HX, DeVico AL, Lewis GK, Williams C, Pinter A, Fong Y, Janes H, DeCamp A, Huang Y, Rao M, Billings E, Karasavvas N, Robb ML, Ngauy V, de Souza MS, Paris R, Ferrari G, Bailer RT, Soderberg KA, Andrews C, Berman PW, Frahm N, De Rosa SC, Alpert MD, Yates NL, Shen X, Koup RA, Pitisuttithum P, Kaewkungwal J, Nitayaphan S, Rerks-Ngarm S, Michael NL, Kim JH 2012 Immune-correlates analysis of an HIV-1 vaccine efficacy trial. N Engl J Med 366:1275–1286. doi:10.1056/NEJMoa1113425. 22475592 PMC3371689

[3] Ma X, Lu M, Gorman J, Terry DS, Hong X, Zhou Z, Zhao H, Altman RB, Arthos J, Blanchard SC, Kwong PD, Munro JB, Mothes W 21 3 2018, posting date HIV-1 Env trimer opens through an asymmetric intermediate in which individual protomers adopt distinct conformations. Elife doi:10.7554/eLife.34271. PMC5896952 29561264

[4] Veillette M, Desormeaux A, Medjahed H, Gharsallah NE, Coutu M, Baalwa J, Guan Y, Lewis G, Ferrari G, Hahn BH, Haynes BF, Robinson JE, Kaufmann DE, Bonsignori M, Sodroski J, Finzi A 2014 Interaction with cellular CD4 exposes HIV-1 envelope epitopes targeted by antibody-dependent cell-mediated cytotoxicity. J Virol 88:2633–2644. doi:10.1128/JVI.03230-13. 24352444 PMC3958102

[5] Yates NL, Liao H-X, Fong Y, deCamp A, Vandergrift NA, Williams WT, Alam SM, Ferrari G, Yang Z-y, Seaton KE, Berman PW, Alpert MD, Evans DT, O'Connell RJ, Francis D, Sinangil F, Lee C, Nitayaphan S, Rerks-Ngarm S, Kaewkungwal J, Pitisuttithum P, Tartaglia J, Pinter A, Zolla-Pazner S, Gilbert PB, Nabel GJ, Michael NL, Kim JH, Montefiori DC, Haynes BF, Tomaras GD 2014 Vaccine-induced Env V1-V2 IgG3 correlates with lower HIV-1 infection risk and declines soon after vaccination. Sci Transl Med 6:228ra39. doi:10.1126/scitranslmed.3007730. PMC4116665 24648342

[6] Tomaras GD, Ferrari G, Shen X, Alam SM, Liao HX, Pollara J, Bonsignori M, Moody MA, Fong Y, Chen X 28 5 2013, posting date Vaccine-induced plasma IgA specific for the C1 region of the HIV-1 envelope blocks binding and effector function of IgG. Proc Natl Acad Sci U S A doi:10.1073/pnas.1301456110. PMC3670311 23661056

[7] Chung AW, Ghebremichael M, Robinson H, Brown E, Choi I, Lane S, Dugast AS, Schoen MK, Rolland M, Suscovich TJ, Mahan AE, Liao L, Streeck H, Andrews C, Rerks-Ngarm S, Nitayaphan S, de Souza MS, Kaewkungwal J, Pitisuttithum P, Francis D, Michael NL, Kim JH, Bailey-Kellogg C, Ackerman ME, Alter G 2014 Polyfunctional Fc-effector profiles mediated by IgG subclass selection distinguish RV144 and VAX003 vaccines. Sci Transl Med 6:228ra38. doi:10.1126/scitranslmed.3007736. 24648341

[8] Checkley MA, Luttge BG, Freed EO 2011 HIV-1 envelope glycoprotein biosynthesis, trafficking, and incorporation. J Mol Biol 410:582–608. doi:10.1016/j.jmb.2011.04.042. 21762802 PMC3139147

[9] Merk A, Subramaniam S 2013 HIV-1 envelope glycoprotein structure. Curr Opin Struct Biol 23:268–276. doi:10.1016/j.sbi.2013.03.007. 23602427 PMC3676719

[10] Wyatt R, Kwong PD, Desjardins E, Sweet RW, Robinson J, Hendrickson WA, Sodroski JG 1998 The antigenic structure of the HIV gp120 envelope glycoprotein. Nature 393:705–711. doi:10.1038/31514. 9641684

[11] Allan JS, Coligan JE, Barin F, McLane MF, Sodroski JG, Rosen CA, Haseltine WA, Lee TH, Essex M 1985 Major glycoprotein antigens that induce antibodies in AIDS patients are encoded by HTLV-III. Science 228:1091–1094. doi:10.1126/science.2986290. 2986290

[12] Robey WG, Safai B, Oroszlan S, Arthur LO, Gonda MA, Gallo RC, Fischinger PJ 1985 Characterization of envelope and core structural gene products of HTLV-III with sera from AIDS patients. Science 228:593–595. doi:10.1126/science.2984774. 2984774

[13] Munro JB, Gorman J, Ma X, Zhou Z, Arthos J, Burton DR, Koff WC, Courter JR, Smith AB III, Kwong PD, Blanchard SC, Mothes W 2014 Conformational dynamics of single HIV-1 envelope trimers on the surface of native virions. Science 346:759–763. doi:10.1126/science.1254426. 25298114 PMC4304640

[14] Prevost J, Richard J, Ding S, Pacheco B, Charlebois R, Hahn BH, Kaufmann DE, Finzi A 2018 Envelope glycoproteins sampling states 2/3 are susceptible to ADCC by sera from HIV-1-infected individuals. Virology 515:38–45. doi:10.1016/j.virol.2017.12.002. 29248757 PMC5843759

[15] Veillette M, Coutu M, Richard J, Batraville LA, Dagher O, Bernard N, Tremblay C, Kaufmann DE, Roger M, Finzi A 2015 The HIV-1 gp120 CD4-bound conformation is preferentially targeted by antibody-dependent cellular cytotoxicity-mediating antibodies in sera from HIV-1-infected individuals. J Virol 89:545–551. doi:10.1128/JVI.02868-14. 25339767 PMC4301108

[16] Richard J, Prevost J, Baxter AE, von Bredow B, Ding S, Medjahed H, Delgado GG, Brassard N, Sturzel CM, Kirchhoff F, Hahn BH, Parsons MS, Kaufmann DE, Evans DT, Finzi A 2018 Uninfected bystander cells impact the measurement of HIV-specific antibody-dependent cellular cytotoxicity responses. mBio 9:e00358-18. doi:10.1128/mBio.00358-18. 29559570 PMC5874913

[17] Richard J, Veillette M, Ding S, Zoubchenok D, Alsahafi N, Coutu M, Brassard N, Park J, Courter JR, Melillo B, Smith AB III, Shaw GM, Hahn BH, Sodroski J, Kaufmann DE, Finzi A 2016 Small CD4 mimetics prevent HIV-1 uninfected bystander CD4+ T cell killing mediated by antibody-dependent cell-mediated cytotoxicity. EBioMedicine 3:122–134. doi:10.1016/j.ebiom.2015.12.004. 26870823 PMC4739418

[18] Guan Y, Pazgier M, Sajadi MM, Kamin-Lewis R, Al-Darmarki S, Flinko R, Lovo E, Wu X, Robinson JE, Seaman MS, Fouts TR, Gallo RC, DeVico AL, Lewis GK 2013 Diverse specificity and effector function among human antibodies to HIV-1 envelope glycoprotein epitopes exposed by CD4 binding. Proc Natl Acad Sci U S A 110:E69–E78. doi:10.1073/pnas.1217609110. 23237851 PMC3538257

[19] Alsahafi N, Bakouche N, Kazemi M, Richard J, Ding S, Bhattacharyya S, Das D, Anand SP, Prevost J, Tolbert WD, Lu H, Medjahed H, Gendron-Lepage G, Ortega Delgado GG, Kirk S, Melillo B, Mothes W, Sodroski J, Smith AB III, Kaufmann DE, Wu X, Pazgier M, Rouiller I, Finzi A, Munro JB 2019 An asymmetric opening of HIV-1 envelope mediates antibody-dependent cellular cytotoxicity. Cell Host Microbe 25:578–587.e5. doi:10.1016/j.chom.2019.03.002. 30974085 PMC6592637

[20] Ding S, Veillette M, Coutu M, Prevost J, Scharf L, Bjorkman PJ, Ferrari G, Robinson JE, Sturzel C, Hahn BH, Sauter D, Kirchhoff F, Lewis GK, Pazgier M, Finzi A 2016 A Highly Conserved Residue of the HIV-1 gp120 Inner Domain Is Important for Antibody-Dependent Cellular Cytotoxicity Responses Mediated by Anti-cluster A Antibodies. J Virol 90:2127–2134. doi:10.1128/JVI.02779-15. 26637462 PMC4733974

[21] Tolbert WD, Gohain N, Veillette M, Chapleau JP, Orlandi C, Visciano ML, Ebadi M, DeVico AL, Fouts TR, Finzi A, Lewis GK, Pazgier M 2016 Paring down HIV Env: design and crystal structure of a stabilized inner domain of HIV-1 gp120 displaying a major ADCC target of the A32 region. Structure 24:697–709. doi:10.1016/j.str.2016.03.005. 27041594 PMC4856543

[22] Finzi A, Xiang SH, Pacheco B, Wang L, Haight J, Kassa A, Danek B, Pancera M, Kwong PD, Sodroski J 2010 Topological layers in the HIV-1 gp120 inner domain regulate gp41 interaction and CD4-triggered conformational transitions. Mol Cell 37:656–667. doi:10.1016/j.molcel.2010.02.012. 20227370 PMC2854584

[23] Alsahafi N, Ding S, Richard J, Markle T, Brassard N, Walker B, Lewis GK, Kaufmann DE, Brockman MA, Finzi A 2015 Nef proteins from HIV-1 elite controllers are inefficient at preventing antibody-dependent cellular cytotoxicity. J Virol 90:2993–3002. doi:10.1128/JVI.02973-15. 26719277 PMC4810628

[24] Pollara J, Hart L, Brewer F, Pickeral J, Packard BZ, Hoxie JA, Komoriya A, Ochsenbauer C, Kappes JC, Roederer M, Huang Y, Weinhold KJ, Tomaras GD, Haynes BF, Montefiori DC, Ferrari G 2011 High-throughput quantitative analysis of HIV-1 and SIV-specific ADCC-mediating antibody responses. Cytometry A 79:603–612. doi:10.1002/cyto.a.21084. 21735545 PMC3692008

[25] Ferrari G, Pollara J, Kozink D, Harms T, Drinker M, Freel S, Moody MA, Alam SM, Tomaras GD, Ochsenbauer C, Kappes JC, Shaw GM, Hoxie JA, Robinson JE, Haynes BF 2011 An HIV-1 gp120 envelope human monoclonal antibody that recognizes a C1 conformational epitope mediates potent antibody-dependent cellular cytotoxicity (ADCC) activity and defines a common ADCC epitope in human HIV-1 serum. J Virol 85:7029–7036. doi:10.1128/JVI.00171-11. 21543485 PMC3126567

[26] Trkola A, Matthews J, Gordon C, Ketas T, Moore JP 1999 A cell line-based neutralization assay for primary human immunodeficiency virus type 1 isolates that use either the CCR5 or the CXCR4 coreceptor. J Virol 73:8966–8974. 10516002 10.1128/jvi.73.11.8966-8974.1999 PMC112928

[27] Howell DN, Andreotti PE, Dawson JR, Cresswell P 1985 Natural killing target antigens as inducers of interferon: studies with an immunoselected, natural killing-resistant human T lymphoblastoid cell line. J Immunol 134:971–976. 3871222

[28] Wyatt R, Moore J, Accola M, Desjardin E, Robinson J, Sodroski J 1995 Involvement of the V1/V2 variable loop structure in the exposure of human immunodeficiency virus type 1 gp120 epitopes induced by receptor binding. J Virol 69:5723–5733. 7543586 10.1128/jvi.69.9.5723-5733.1995 PMC189432

[29] Moore JP, Thali M, Jameson BA, Vignaux F, Lewis GK, Poon SW, Charles M, Fung MS, Sun B, Durda PJ 1993 Immunochemical analysis of the gp120 surface glycoprotein of human immunodeficiency virus type 1: probing the structure of the C4 and V4 domains and the interaction of the C4 domain with the V3 loop. J Virol 67:4785–4796. 7687303 10.1128/jvi.67.8.4785-4796.1993 PMC237865

[30] von Bredow B, Arias JF, Heyer LN, Moldt B, Le K, Robinson JE, Zolla-Pazner S, Burton DR, Evans DT 2016 Comparison of antibody-dependent cell-mediated cytotoxicity and virus neutralization by HIV-1 Env-specific monoclonal antibodies. J Virol 90:6127–6139. doi:10.1128/JVI.00347-16. 27122574 PMC4907221

[31] Bruel T, Guivel-Benhassine F, Amraoui S, Malbec M, Richard L, Bourdic K, Donahue DA, Lorin V, Casartelli N, Noel N, Lambotte O, Mouquet H, Schwartz O 2016 Elimination of HIV-1-infected cells by broadly neutralizing antibodies. Nat Commun 7:10844. doi:10.1038/ncomms10844. 26936020 PMC4782064

[32] Bruel T, Guivel-Benhassine F, Lorin V, Lortat-Jacob H, Baleux F, Bourdic K, Noel N, Lambotte O, Mouquet H, Schwartz O 29 3 2017, posting date Lack of ADCC breadth of human nonneutralizing anti-HIV-1 antibodies. J Virol doi:10.1128/JVI.02440-16. PMC5375671 28122982

[33] Lee WS, Prevost J, Richard J, van der Sluis RM, Lewin SR, Pazgier M, Finzi A, Parsons MS, Kent SJ 1 5 2019, posting date CD4- and time-dependent susceptibility of HIV-1-infected cells to ADCC. J Virol doi:10.1128/JVI.01901-18. PMC6498039 30842324

[34] Prevost J, Richard J, Medjahed H, Alexander A, Jones J, Kappes JC, Ochsenbauer C, Finzi A 13 6 2018, posting date Incomplete downregulation of CD4 expression affects HIV-1 Env conformation and antibody-dependent cellular cytotoxicity responses. J Virol doi:10.1128/JVI.00484-18. PMC6002730 29669829

[35] Bonsignori M, Pollara J, Moody MA, Alpert MD, Chen X, Hwang KK, Gilbert PB, Huang Y, Gurley TC, Kozink DM, Marshall DJ, Whitesides JF, Tsao CY, Kaewkungwal J, Nitayaphan S, Pitisuttithum P, Rerks-Ngarm S, Kim JH, Michael NL, Tomaras GD, Montefiori DC, Lewis GK, DeVico A, Evans DT, Ferrari G, Liao HX, Haynes BF 2012 Antibody-dependent cellular cytotoxicity-mediating antibodies from an HIV-1 vaccine efficacy trial target multiple epitopes and preferentially use the VH1 gene family. J Virol 86:11521–11532. doi:10.1128/JVI.01023-12. 22896626 PMC3486290

[36] Packard BZ, Telford WG, Komoriya A, Henkart PA 2007 Granzyme B activity in target cells detects attack by cytotoxic lymphocytes. J Immunol 179:3812–3820. doi:10.4049/jimmunol.179.6.3812. 17785818

[37] Richard J, Prevost J, Alsahafi N, Ding S, Finzi A 2018 Impact of HIV-1 envelope conformation on ADCC responses. Trends Microbiol 26:253–265. doi:10.1016/j.tim.2017.10.007. 29162391

[38] Imbeault M, Lodge R, Ouellet M, Tremblay MJ 2009 Efficient magnetic bead-based separation of HIV-1-infected cells using an improved reporter virus system reveals that p53 up-regulation occurs exclusively in the virus-expressing cell population. Virology 393:160–167. doi:10.1016/j.virol.2009.07.009. 19692106

[39] Calarese DA, Scanlan CN, Zwick MB, Deechongkit S, Mimura Y, Kunert R, Zhu P, Wormald MR, Stanfield RL, Roux KH, Kelly JW, Rudd PM, Dwek RA, Katinger H, Burton DR, Wilson IA 2003 Antibody domain exchange is an immunological solution to carbohydrate cluster recognition. Science 300:2065–2071. doi:10.1126/science.1083182. 12829775

[40] West AP Jr, Galimidi RP, Foglesong CP, Gnanapragasam PN, Huey-Tubman KE, Klein JS, Suzuki MD, Tiangco NE, Vielmetter J, Bjorkman PJ 2009 Design and expression of a dimeric form of human immunodeficiency virus type 1 antibody 2G12 with increased neutralization potency. J Virol 83:98–104. doi:10.1128/JVI.01564-08. 18945777 PMC2612297

[41] Lu M, Ma X, Castillo-Menendez LR, Gorman J, Alsahafi N, Ermel U, Terry DS, Chambers M, Peng D, Zhang B, Zhou T, Reichard N, Wang K, Grover JR, Carman BP, Gardner MR, Nikic-Spiegel I, Sugawara A, Arthos J, Lemke EA, Smith AB III, Farzan M, Abrams C, Munro JB, McDermott AB, Finzi A, Kwong PD, Blanchard SC, Sodroski JG, Mothes W 2019 Associating HIV-1 envelope glycoprotein structures with states on the virus observed by smFRET. Nature 568:415–419. doi:10.1038/s41586-019-1101-y. 30971821 PMC6655592

[42] Gomez-Roman VR, Florese RH, Patterson LJ, Peng B, Venzon D, Aldrich K, Robert-Guroff M 2006 A simplified method for the rapid fluorometric assessment of antibody-dependent cell-mediated cytotoxicity. J Immunol Methods 308:53–67. doi:10.1016/j.jim.2005.09.018. 16343526

[43] Kramski M, Schorcht A, Johnston AP, Lichtfuss GF, Jegaskanda S, De Rose R, Stratov I, Kelleher AD, French MA, Center RJ, Jaworowski A, Kent SJ 2012 Role of monocytes in mediating HIV-specific antibody-dependent cellular cytotoxicity. J Immunol Methods 384:51–61. doi:10.1016/j.jim.2012.07.006. 22841577

[44] Mengistu M, Tang AH, Foulke JS Jr, Blanpied TA, Gonzalez MW, Spouge JL, Gallo RC, Lewis GK, DeVico AL 2017 Patterns of conserved gp120 epitope presentation on attached HIV-1 virions. Proc Natl Acad Sci U S A 114:E9893–E9902. doi:10.1073/pnas.1705074114. 29087304 PMC5699032

[45] Volchkov VE, Becker S, Volchkova VA, Ternovoj VA, Kotov AN, Netesov SV, Klenk HD 1995 GP mRNA of Ebola virus is edited by the Ebola virus polymerase and by T7 and vaccinia virus polymerases. Virology 214:421–430. doi:10.1006/viro.1995.0052. 8553543

[46] Volchkov VE, Feldmann H, Volchkova VA, Klenk HD 1998 Processing of the Ebola virus glycoprotein by the proprotein convertase furin. Proc Natl Acad Sci U S A 95:5762–5767. doi:10.1073/pnas.95.10.5762. 9576958 PMC20453

[47] Bukreyev A, Yang L, Collins PL 2012 The secreted G protein of human respiratory syncytial virus antagonizes antibody-mediated restriction of replication involving macrophages and complement. J Virol 86:10880–10884. doi:10.1128/JVI.01162-12. 22837211 PMC3457292

[48] Bukreyev A, Yang L, Fricke J, Cheng L, Ward JM, Murphy BR, Collins PL 2008 The secreted form of respiratory syncytial virus G glycoprotein helps the virus evade antibody-mediated restriction of replication by acting as an antigen decoy and through effects on Fc receptor-bearing leukocytes. J Virol 82:12191–12204. doi:10.1128/JVI.01604-08. 18842713 PMC2593351

[49] Anand SP, Finzi A 30 8 2019, posting date Understudied factors influencing Fc-mediated immune responses against viral infections. Vaccines (Basel) doi:10.3390/vaccines7030103. PMC6789852 31480293

[50] Song R, Lisovsky I, Lebouche B, Routy JP, Bruneau J, Bernard NF 2014 HIV protective KIR3DL1/S1-HLA-B genotypes influence NK cell-mediated inhibition of HIV replication in autologous CD4 targets. PLoS Pathog 10:e1003867. doi:10.1371/journal.ppat.1003867. 24453969 PMC3894215

[51] Boulassel MR, Spurll G, Rouleau D, Tremblay C, Edwardes M, Sekaly RP, Lalonde R, Routy JP 2003 Changes in immunological and virological parameters in HIV-1 infected subjects following leukapheresis. J Clin Apher 18:55–60. doi:10.1002/jca.10051. 12874816

[52] Lyerly HK, Reed DL, Matthews TJ, Langlois AJ, Ahearne PA, Petteway SR Jr, Weinhold KJ 1987 Anti-GP 120 antibodies from HIV seropositive individuals mediate broadly reactive anti-HIV ADCC. AIDS Res Hum Retroviruses 3:409–422. doi:10.1089/aid.1987.3.409. 2833917

[53] Cummins LM, Weinhold KJ, Matthews TJ, Langlois AJ, Perno CF, Condie RM, Allain JP 1991 Preparation and characterization of an intravenous solution of IgG from human immunodeficiency virus-seropositive donors. Blood 77:1111–1117. doi:10.1182/blood.V77.5.1111.1111. 1995097

[54] Tremblay-McLean A, Coenraads S, Kiani Z, Dupuy FP, Bernard NF 2019 Expression of ligands for activating natural killer cell receptors on cell lines commonly used to assess natural killer cell function. BMC Immunol 20:8. doi:10.1186/s12865-018-0272-x. 30696399 PMC6352444

[55] Kant S, Zhang N, Routy JP, Tremblay C, Thomas R, Szabo J, Cote P, Trottier B, LeBlanc R, Rouleau D, Harris M, Dupuy FP, Bernard NF 2019 Quantifying anti-HIV envelope-specific antibodies in plasma from HIV infected individuals. Viruses 11:487. doi:10.3390/v11060487. PMC6631318 31141927

[56] Isitman G, Lisovsky I, Tremblay-McLean A, Parsons MS, Shoukry NH, Wainberg MA, Bruneau J, Bernard NF 2015 Natural killer cell education does not affect the magnitude of granzyme B delivery to target cells by antibody-dependent cellular cytotoxicity. AIDS 29:1433–1443. doi:10.1097/QAD.0000000000000729. 26244383

[57] Lisovsky I, Kant S, Tremblay-McLean A, Isitman G, Kiani Z, Dupuy FP, Gilbert L, Bruneau J, Shoukry NH, Lebouché B, Bernard NF 2019 Differential contribution of education through KIR2DL1, KIR2DL3, and KIR3DL1 to antibody-dependent (AD) NK cell activation and ADCC. J Leukoc Biol 105:551. doi:10.1002/JLB.4A0617-242RRR. 30698860 PMC6916277

[58] Burton DR, Pyati J, Koduri R, Sharp SJ, Thornton GB, Parren PW, Sawyer LS, Hendry RM, Dunlop N, Nara PL 1994 Efficient neutralization of primary isolates of HIV-1 by a recombinant human monoclonal antibody. Science 266:1024–1027. doi:10.1126/science.7973652. 7973652

[59] Roben P, Moore JP, Thali M, Sodroski J, Barbas CF III, Burton DR 1994 Recognition properties of a panel of human recombinant Fab fragments to the CD4 binding site of gp120 that show differing abilities to neutralize human immunodeficiency virus type 1. J Virol 68:4821–4828. 7518527 10.1128/jvi.68.8.4821-4828.1994 PMC236421

[60] Barbas CF III, Bjorling E, Chiodi F, Dunlop N, Cababa D, Jones TM, Zebedee SL, Persson MA, Nara PL, Norrby E 1992 Recombinant human Fab fragments neutralize human type 1 immunodeficiency virus in vitro. Proc Natl Acad Sci U S A 89:9339–9343. doi:10.1073/pnas.89.19.9339. 1384050 PMC50122

[61] Burton DR, Barbas CF III, Persson MA, Koenig S, Chanock RM, Lerner RA 1991 A large array of human monoclonal antibodies to type 1 human immunodeficiency virus from combinatorial libraries of asymptomatic seropositive individuals. Proc Natl Acad Sci U S A 88:10134–10137. doi:10.1073/pnas.88.22.10134. 1719545 PMC52882

[62] Wu X, Yang Z-Y, Li Y, Hogerkorp C-M, Schief WR, Seaman MS, Zhou T, Schmidt SD, Wu L, Xu L, Longo NS, McKee K, O'Dell S, Louder MK, Wycuff DL, Feng Y, Nason M, Doria-Rose N, Connors M, Kwong PD, Roederer M, Wyatt RT, Nabel GJ, Mascola JR 2010 Rational design of envelope identifies broadly neutralizing human monoclonal antibodies to HIV-1. Science 329:856–861. doi:10.1126/science.1187659. 20616233 PMC2965066

[63] Diskin R, Scheid JF, Marcovecchio PM, West AP Jr, Klein F, Gao H, Gnanapragasam PN, Abadir A, Seaman MS, Nussenzweig MC, Bjorkman PJ 2011 Increasing the potency and breadth of an HIV antibody by using structure-based rational design. Science 334:1289–1293. doi:10.1126/science.1213782. 22033520 PMC3232316

[64] Shingai M, Nishimura Y, Klein F, Mouquet H, Donau OK, Plishka R, Buckler-White A, Seaman M, Piatak M Jr, Lifson JD, Dimitrov DS, Nussenzweig MC, Martin MA 2013 Antibody-mediated immunotherapy of macaques chronically infected with SHIV suppresses viraemia. Nature 503:277–280. doi:10.1038/nature12746. 24172896 PMC4133787

[65] Moore JP, Sattentau QJ, Wyatt R, Sodroski J 1994 Probing the structure of the human immunodeficiency virus surface glycoprotein gp120 with a panel of monoclonal antibodies. J Virol 68:469–484. 7504741 10.1128/jvi.68.1.469-484.1994 PMC236308

[66] Tolbert WD, Gohain N, Alsahafi N, Van V, Orlandi C, Ding S, Martin L, Finzi A, Lewis GK, Ray K, Pazgier M 2017 Targeting the late stage of HIV-1 entry for antibody-dependent cellular cytotoxicity: structural basis for Env epitopes in the C11 region. Structure 25:1719–1731.e4. doi:10.1016/j.str.2017.09.009. 29056481 PMC5677539

[67] Gohain N, Tolbert WD, Orlandi C, Richard J, Ding S, Chen X, Bonsor DA, Sundberg EJ, Lu W, Ray K, Finzi A, Lewis GK, Pazgier M 2016 Molecular basis for epitope recognition by non-neutralizing anti-gp41 antibody F240. Sci Rep 6:36685. doi:10.1038/srep36685. 27827447 PMC5101508

[68] Walker LM, Phogat SK, Chan-Hui PY, Wagner D, Phung P, Goss JL, Wrin T, Simek MD, Fling S, Mitcham JL, Lehrman JK, Priddy FH, Olsen OA, Frey SM, Hammond PW, Protocol G Principal Investigators, Kaminsky S, Zamb T, Moyle M, Koff WC, Poignard P, Burton DR 2009 Broad and potent neutralizing antibodies from an African donor reveal a new HIV-1 vaccine target. Science 326:285–289. doi:10.1126/science.1178746. 19729618 PMC3335270

[69] Walker LM, Huber M, Doores KJ, Falkowska E, Pejchal R, Julien JP, Wang SK, Ramos A, Chan-Hui PY, Moyle M, Mitcham JL, Hammond PW, Olsen OA, Phung P, Fling S, Wong CH, Phogat S, Wrin T, Simek MD, Protocol G Principal Investigators, Koff WC, Wilson IA, Burton DR, Poignard P 2011 Broad neutralization coverage of HIV by multiple highly potent antibodies. Nature 477:466–470. doi:10.1038/nature10373. 21849977 PMC3393110

[70] Buchacher A, Predl R, Strutzenberger K, Steinfellner W, Trkola A, Purtscher M, Gruber G, Tauer C, Steindl F, Jungbauer A 1994 Generation of human monoclonal antibodies against HIV-1 proteins; electrofusion and Epstein-Barr virus transformation for peripheral blood lymphocyte immortalization. AIDS Res Hum Retroviruses 10:359–369. doi:10.1089/aid.1994.10.359. 7520721

[71] Crawford JM, Earl PL, Moss B, Reimann KA, Wyand MS, Manson KH, Bilska M, Zhou JT, Pauza CD, Parren PW, Burton DR, Sodroski JG, Letvin NL, Montefiori DC 1999 Characterization of primary isolate-like variants of simian-human immunodeficiency virus. J Virol 73:10199–10207. 10559336 10.1128/jvi.73.12.10199-10207.1999 PMC113073

[72] Etemad-Moghadam B, Sun Y, Nicholson EK, Karlsson GB, Schenten D, Sodroski J 1999 Determinants of neutralization resistance in the envelope glycoproteins of a simian-human immunodeficiency virus passaged in vivo. J Virol 73:8873–8879. 10482646 10.1128/jvi.73.10.8873-8879.1999 PMC112913

[73] Mascola JR, Lewis MG, Stiegler G, Harris D, VanCott TC, Hayes D, Louder MK, Brown CR, Sapan CV, Frankel SS, Lu Y, Robb ML, Katinger H, Birx DL 1999 Protection of macaques against pathogenic simian/human immunodeficiency virus 89.6PD by passive transfer of neutralizing antibodies. J Virol 73:4009–4018. 10196297 10.1128/jvi.73.5.4009-4018.1999 PMC104180

